# Dextran sulfate from *Leuconostoc mesenteroides* B512F exerts potent antiviral activity against SARS-CoV-2 *in vitro* and *in vivo*

**DOI:** 10.3389/fmicb.2023.1185504

**Published:** 2023-05-03

**Authors:** Sabina Andreu, Cayetano von Kobbe, Pilar Delgado, Inés Ripa, María José Buzón, Meritxell Genescà, Núria Gironès, Javier del Moral-Salmoral, Gustavo A. Ramírez, Sonia Zúñiga, Luis Enjuanes, José Antonio López-Guerrero, Raquel Bello-Morales

**Affiliations:** ^1^Department of Molecular Biology, Universidad Autónoma de Madrid, Madrid, Spain; ^2^Centro de Biología Molecular Severo Ochoa, Spanish National Research Council—Universidad Autónoma de Madrid (CSIC-UAM), Madrid, Spain; ^3^Infectious Diseases Department, Vall d'Hebron Research Institute (VHIR), Hospital Universitari Vall d'Hebron, Universitat Autònoma de Barcelona, VHIR Task Force COVID-19, Barcelona, Spain; ^4^Animal Science Department, University of Lleida (UDL), Lleida, Spain; ^5^Department of Molecular and Cell Biology, Centro Nacional de Biotecnología-Consejo Superior de Investigaciones Científicas (CNB-CSIC), Madrid, Spain

**Keywords:** virology, SARS–CoV–2, dextran sulfate, antivirals, nebulization

## Abstract

The emergent human coronavirus SARS-CoV-2 and its resistance to current drugs makes the need for new potent treatments for COVID-19 patients strongly necessary. Dextran sulfate (DS) polysaccharides have long demonstrated antiviral activity against different enveloped viruses *in vitro*. However, their poor bioavailability has led to their abandonment as antiviral candidates. Here, we report for the first time the broad-spectrum antiviral activity of a DS-based extrapolymeric substance produced by the lactic acid bacterium *Leuconostoc mesenteroides* B512F. Time of addition assays with SARS-CoV-2 pseudoviruses in *in vitro* models confirm the inhibitory activity of DSs in the early stages of viral infection (viral entry). In addition, this exopolysaccharide substance also reports broad-spectrum antiviral activity against several enveloped viruses such as SARS-CoV-2, HCoV229E, HSV-1, in *in vitro* models and in human lung tissue. The toxicity and antiviral capacity of DS from *L. mesenteroides* was tested *in vivo* in mouse models which are susceptible to SARS-CoV-2 infection. The described DS, administered by inhalation, a new route of administration for these types of polymers, shows strong inhibition of SARS-CoV-2 infection *in vivo*, significantly reducing animal mortality and morbidity at non-toxic doses. Therefore, we suggest that it may be considered as a potential candidate for antiviral therapy against SARS-CoV-2.

## 1. Introduction

Several microorganisms, both prokaryotic and eukaryotic, can produce and secrete to the environment extracellular polymeric substances (EPSs) (Bello-Morales et al., 2022), highly heterogeneous and variable polymers composed by carbohydrates, proteins, lipids, nucleic acids and humic substances (More et al., 2014; Bello-Morales et al., 2022). EPSs may perform important adaptive functions, including protection from adverse external conditions and attachment to surfaces leading to the formation of biofilms (Flemming et al., 2016; Costa et al., 2018). EPSs can also exert antimicrobial activity (Poli et al., 2010; Xiao and Zheng, 2016), and the antiviral effect of these substances against several viruses, including herpes simplex virus type 1 (HSV-1) (Marino-Merlo et al., 2017; Sánchez-León et al., 2020), herpes simplex virus type 2 (HSV-2) (Arena et al., 2005), or influenza virus (Zheng et al., 2006), has been reported.

The antiviral effect of sulfated polysaccharides and other polyanions has been known for decades (Witvrouw et al., 1994). Initially, HSV-1 was inhibited by heparin and other related polyanions (Nahmias and Kibrick, 1964; Nahmias et al., 1964; Takemoto and Fabisch, 1964; Vaheri, 1964). Then, several polysulfates were demonstrated to have a high inhibitory effect against human immunodeficiency virus (HIV) in cell culture (Ito et al., 1987; Baba et al., 1988a; Bagasra and Lischner, 1988; Handa et al., 1991; Witvrouw et al., 1994). Other enveloped viruses, including HSV-2, influenza A virus, respiratory syncytial virus (RSV), cytomegalovirus (CMV), vesicular stomatitis virus (VSV), Sindbis virus, Semliki Forest virus and arenaviruses were also proven to be highly susceptible to polyanions *in vitro* (Baba et al., 1988b; Andrei and De Clercq, 1990; Mastromarino et al., 1991; Lüscher-Mattli et al., 1993; Schols et al., 2016; Sánchez-León et al., 2020; Bello-Morales et al., 2022). Furthermore, a recent study has demonstrated that dextran sulfate (DS), a branched gluocpolysaccharide produced by lactic acid bacteria, inhibits infection of a SARS-CoV-2-pseudotyped HIV-1-based vector *in vitro* (Izumida et al., 2022). These findings generated great initial hope, since, besides their potent antiviral capacity, sulfated polysaccharides were non-toxic in animals and non-specific, so they might be used against different variants or even different viruses.

However, early expectations were followed by wide skepticism when *in vivo* studies showed a poor bioavailability after oral and intravenous administration (Witvrouw et al., 1994). Nevertheless, we still consider that polyanions might be a promising clinical strategy as antivirals against enveloped viruses (Bello-Morales et al., 2022). The key question is how to administer them. Although the poor bioavailability revealed by studies on drug administration led to abandon them as antiviral candidates, we proposed that this difficulty could be overcome by the use of other administration strategies, such as nebulization of aerosols to reach the low respiratory tract (Bello-Morales et al., 2022).

The zoonotic COVID-19 pandemic arisen in late 2019 posed a serious threat to global health and economy. The severe acute respiratory syndrome coronavirus 2 (SARS-CoV-2) (Gorbalenya et al., 2020), the causal agent for this coronavirus disease, has been responsible for millions of infections and deaths. To date, the World Health Organization (WHO), has reported more than 633 million confirmed cases of this disease in the world, including more than 6.6 million deaths [World Health Organization (WHO), 2022]. Two other zoonotic coronaviruses have also caused fatal disease in humans in the last two decades: the severe acute respiratory syndrome coronavirus (SARS-CoV), emerged in China in 2002, and the Middle East respiratory syndrome coronavirus (MERS-CoV), appeared in the Middle East in 2012 (Enjuanes et al., 2016; Choudhary et al., 2021).

Regarding COVID-19 therapeutics, science and technology have come together to produce numerous vaccines in record time (Sarangi et al., 2021). However, besides prevention, the lack of efficient drugs to treat COVID-19 and other respiratory viruses makes imperative to continue the search for useful antiviral agents to treat this kind of viruses. Here we report for the first time the antiviral effect of a dextran sulfate (DS) from *Leuconostoc mesenteroides* B512F against SARS-CoV-2 *in vitro* and *in vivo*. In this work, antiviral assays in mice have been carried out applying the exopolymer by inhalation, a novel administration route for sulfated polyanions. Unlike previous reports using other administration strategies, our results show antiviral effect in mice treated with this inhaled dextran sulfate. This result opens a promising clinical alternative for treatment of infections produced by SARS-CoV-2 and other respiratory viruses.

## 2. Materials and methods

### 2.1. Cell lines

The Vero cell line, derived from the kidney of an adult African green monkey, was kindly provided by Dr. Enrique Tabarés (UAM, Madrid, Spain). The Huh-7 cell line (Nakabayashi et al., 1982) was generously provided by Dr. Sonia Zúñiga (CNB-CSIC, Madrid, Spain). HeLa cells (CCL-2) and Vero-E6 cells (CRL-1586) were purchased from the American Tissue Culture Collection (ATCC). Human embryonic kidney HEK293T cells native or expressing human ACE2 were generated by lentiviral transduction with vector CSIB and selection in blasticidin S (Horndler et al., 2021). All cell lines were routinely tested for the absence of mycoplasma.

Cell lines were cultured in low-glucose Dulbecco's modified Eagle medium (DMEM) (Life Technologies) supplemented with 5% fetal bovine serum (FBS), penicillin (50 U/mL) and streptomycin (50 μg/mL) at 37°C in a humidified atmosphere of 5% CO_2_.

### 2.2. Lung tissue

Lung tissues were obtained from patients with no history of COVID-19 and with a recent negative PCR test for SARS-CoV-2 infection undergoing thoracic surgical resection at the Thoracic Surgery Service of the Vall d'Hebron University Hospital (Barcelona, Spain). Cell extraction was performed as described in Grau-Expósito et al. (2022). Briefly, non-neoplastic tissue areas were dissected into small blocks and digested with collagenase IV (Gibco) and DNase I (Roche) for 30 min at 37°C and 400 rpm, and mechanically digested with a pestle. The resulted cellular suspension was subjected to several filtrations and washes with PBS and finally resuspended with RPMI 1640 supplemented with 5% FBS, 100 U/ml penicillin and 100 ug/ml streptomycin. Cell number and viability were evaluated with the LUNA Automated Cell Counter (Logos Biosystems).

### 2.3. Viruses

HCoV-229E expressing a GFP reporter protein was generously provided by Dr. Volker Thiel, from the University of Bern. This virus was propagated on Huh-7 cells for 5 days at 33°C with 5% CO_2_. The infectious titer of the virus stocks was determined according to the Reed and Muench formula (Reed and Muench, 1938) on Huh-7 cell monolayers by the endpoint dilution assay described in Andreu et al. (2021). HSV-1 K26-GFP (a kind gift from. Dr. Prashant Desai; Johns Hopkins University, Baltimore, USA) was obtained by fusion of green fluorescent protein (GFP) with HSV-1 capsid protein VP26 (Desai and Person, 1998). K26-GFP was propagated and titrated in Vero cells. Minute virus of mice (MVM, prototype strain) (Crawford, 1966), which can infect human tumor cells (Riolobos et al., 2010) was kindly provided by Dr. José M. Almendral (CBMSO, Madrid, Spain). SARS-CoV-2 virus (isolate Navarra-2473) was obtained from the nasal sample of a COVID-19 patient admitted to the University of Navarra Clinic (Pamplona, Spain) (Maestro et al., 2021), and was gently provided by Dr. Cristian Smerdou (CIMA, Universidad de Navarra, Spain). The SARS-CoV-2 strain NL/2020 was provided by Pablo Gastaminza (CNB-CSIC, Madrid, Spain).

Lentiviral particles expressing either SARS-CoV-2 spike (St) protein (Wuhan, truncated) or vesicular stomatitis virus (VSV) protein and GFP reporter protein were generated as in Horndler et al. (2021). Briefly, pseudoviruses were obtained by co-transfecting plasmids pCMVA (gag/pol), p-HR-SIN-GFP and either a truncated S envelope (pCR3.1-St) or VSV envelope (pMD2.G) using the JetPEI transfection reagent (Polyplus Transfection). Viral supernatants were obtained after 24 and 48 h of transfection and pooled. Polybrene (4 μg/ml) was added to the viral supernatants before the addition to ACE2+HEK293T cells. Cells were centrifuged for 70 min at 2,100 rpm at 32°C and left in culture for 48 h. Finally, cells were resuspended with 5 mM EDTA and fixed for flow cytometry analysis. Both LV-St and LV-VSV were titrated on ACE2+HEK293T cells by analysis of GFP+ cells on a FACSCanto™ II Flow Cytometer (Becton-Dickinson), and data were processed with FlowJo software (BD, version 10.6.2).

SARS-CoV-2 spike pseudotyped VSV^*^ΔG(Luc)-S was generated following the protocol previously described in Grau-Expósito et al. (2022).

### 2.4. Reagents

Chondroitin sulfate sodium salt from shark cartilage (C4384, P1), dextran sulfate sodium salt from *Leuconostoc mesenteroides* B512F M_w_ >500,000 Da (D8906, P2), dextran sulfate sodium salt M_w_ 7,000-20,000 Da (D51227, P3), and dextran sulfate sodium salt M_r_ ~40,000 Da (42867, P4) were purchased from Sigma-Aldrich (Supplementary Figure 1). All were diluted in water to the stock concentrations and stored at 4°C. Such reagents were used in the cytotoxicity and antiviral assays. P2 was the only one that was subjected to SARS-CoV-2 *in vivo* in mouse models.

The different molecular weight polymers were produced by limited hydrolysis and fractionation. The dextran sulfate P2 was obtained from bacterial culture and subsequent chemical transformation. Regarding P2, fractionation of dextran was performed by ethanol, in which the largest molecular weight dextrans precipitate first. Esterification with sulfuric acid was carried out under mild conditions. The dextran sulfate P2 presents an off-white color appearance and comes in powder form. It has a molecular weight >500,000 Da (dextran starting material) and the sulfur content of the polymer is 16.9% (measured by S/C relation analysis), which is equivalent to ~2.3 sulfate groups per glucosyl residue. The pH of the polymer (1% in water at 25°C) is 7.3 and the solubility in water is 100 mg/ml, being purity >95%. When solubilized, its color changes to a very faint yellow.

### 2.5. Analysis of cell viability

The cytotoxicity of the polymers in Huh-7, ACE2+HEK293T, Vero, Vero E6, and HeLa cell lines was quantified using a CellTiter 96 Aqueous Non-Radioactive Cell Proliferation Assay Kit (Promega) based on MTT reagent. Non-confluent monolayers of cells plated in 96-well tissue culture plates were grown for 24 h before use. Cells were then treated for 48 h with P1, P2, P3, and P4 at concentrations ranging from 0 to 1,000 μg/ml. Four replicates were performed for each concentration. The cells were then incubated as indicated by the manufacturer of the kit, and the resulting colored solution was quantified using the scanning multiwell spectrophotometer iMarkTM Microplate Reader (BioRad), measuring the absorbance at 595 nm. The readouts obtained from the MTT assay were further normalized to the value of untreated cells, and CC_50_ values were calculated.

### 2.6. Viral assays in cell lines

#### 2.6.1. Time of addition experiments

Time of addition experiments with HSV-1 K26 GFP and pseudoviruses LV-St and LV-VSV were performed to study the phase of infection at which candidate compounds exerted their antiviral activity. Vero and ACE2+HEK293T cell cultures were grown in 48-well culture plates and inoculated at a MOI of 0.1 with HSV-1 K26-GFP or pseudoviruses, respectively for 1 h in the presence or absence of the compounds at a temperature of 37°C. Several protocols were tested in which candidate compounds were added before, during, or/and after viral infection (Figure 2A). After 1 h of adsorption, the virus was washed and replaced with fresh 5% FBS complete medium containing or not the tested compounds. At 24 hours post-infection (h p.i.), cells were fixed for flow cytometry analysis.

#### 2.6.2. Antiviral assays with HCoV-229E, HSV-1 K26 GFP, and MVM

Hereunder, the antiviral activity of the polymers (in contact with cell cultures at all times) was assayed. Cells were seeded in 48-well culture plates and treated for 1 h with either P1, P2, P3, or P4 at a range of concentrations between 0 and 1,000 μg/ml. Then, each cell line was infected with its corresponding virus at a specific MOI in the presence of the candidate compounds. Subsequently, the virus was removed, and cells were washed with PBS and maintained in a fresh culture medium containing the polymers, in a humidified atmosphere. Cells were fixed for flow cytometry at different h p.i., according to the virus used: (i) HSV-1 K26-GFP infection (MOI 0.1) in Vero cells at 37°C; samples were collected at 24 h p.i; (ii) HCoV-229E infection (MOI 0.5) in Huh-7 cells at 33°C; samples were gathered at 48 h p.i.; (iii) MVM infection (MOI 0.5) in HeLa cells at 37°C; samples were collected at 24 h p.i.

#### 2.6.3. Antiviral assays with LV-St and LV-VSV pseudoviruses

As for pseudoviruses assays, ACE2+HEK293T cells were seeded in 96-well tissue culture plates and infected with either LV-St or LV-VSV (MOI 0.1) previously mixed for 1 h with the compounds at different concentrations, and in the presence of polybrene (4 μg/ml), and transduced as previously described (Section 2). At 48 h p.i., cells were fixed for flow cytometry.

#### 2.6.4. Antiviral assays with SARS-CoV-2

SARS-CoV-2 *in vitro* infection experiments were performed by the CNB Antiviral Screening Platform using the methodology described in Fàbrega-Ferrer et al. (2022). Briefly, Vero-E6 cells were inoculated with SARS-CoV-2 (strain NL/2020) at a MOI of 0.01 in the presence of the P1, P2, P3, and P4. Remdesivir (RMDV) was used as a positive control (Pruijssers et al., 2020). At 48 h p.i., cells were fixed for 20 min at room temperature with a 4% formaldehyde solution in PBS, washed twice with PBS and incubated with incubation buffer (3% BSA; 0.3% Triton X100 in PBS) for 1 h. A monoclonal antibody against the N protein was diluted in the incubation buffer (1:2000, v/v; Genetex HL344) and incubated with the cells for 1 h; after this time, cells were washed with PBS and subsequently incubated with a 1:500 (v/v) dilution of a goat anti-rabbit conjugated to Alexa 488 (Invitrogen). To control for unexpected toxicity of the compounds, nuclei were stained with DAPI (Life Technologies) during the secondary antibody incubation as recommended by the manufacturer. Cells were washed with PBS and imaged using an automated multimode reader (TECAN Spark Cyto) (Fàbrega-Ferrer et al., 2022). All data are referred to controls where infection efficiency was determined in the presence of the vehicle.

### 2.7. Viral assays in lung tissue

Duplicates of five-fold serial dilutions of the four polymers were tested in human lung tissue (HLT) cells using at least three different donors. HLT cells were added at a density of 300,000 cells/well and incubated with the compounds for 1 h before infection. Then, a MOI of 0.1 of the VSV^*^ΔG(Luc)-S virus was added to the wells, and plates were spinoculated at 1,200 g and 37°C for 2 h. After the infection, fresh RPMI medium was added to the wells and cell suspensions were transferred into a 96-well flat-bottom plate. Cells were then cultured overnight at 37°C in a 5% CO_2_ incubator. Each plate contained the following controls: no cells (background control), cells treated with medium (mock infection), cells infected but untreated (infection control) and cells infected and treated with the drug camostat mesylate (S2874, Sigma) as a positive control (Grau-Expósito et al., 2022). After 20 h, cells were incubated with Britelite plus reagent (Britelite plus kit; PerkinElmer) and then transferred to an opaque black plate. Luminescence was immediately recorded by a luminescence plate reader (LUMIstar Omega). In parallel, drug cytotoxicity was monitored by luminescence. To evaluate cytotoxicity, the CellTiter-Glo Luminescent kit (Promega), was used. Data were normalized to the mock-infected control, after which EC_50_ and CC_50_ values were calculated.

### 2.8. Immunofluorescence microscopy

After viral assays and infections, cells grown on glass coverslips were fixed in 4% paraformaldehyde for 20 min and rinsed with PBS. All cells were then permeabilized with 0.2% Triton X-100, rinsed, and incubated for 30 min at room temperature with incubation buffer. For MVM infected HeLa cell samples, a rabbit polyclonal antiserum against the VP2 N-terminal domain was used for 1 h of incubation (Maroto et al., 2004). Subsequently, an anti-rabbit antibody conjugated to Alexa 488 (Invitrogen) was added for another 1 h. Nuclei were stained with DAPI for 10 min. After thorough washing, coverslips were mounted in Mowiol and imaged using the LSM 710 Inverted Confocal Microscope (Zeiss). Processing of confocal images was performed using the Fiji-ImageJ software (version Image J 1.53c).

### 2.9. Flow cytometry analysis

To perform FACS analysis, cells were dissociated by 1 min incubation with 0.05% trypsin/0.1% EDTA (Invitrogen) at room temperature and washed and fixed in 4% paraformaldehyde for 15 min. Finally, cells were rinsed and resuspended in PBS. Cells were analyzed using a FACSCalibur Flow Cytometer (BD). Data were processed with FlowJo software (BD, version 10.6.2).

### 2.10. *In vivo* toxicity evaluation of polymer 2

To evaluate the toxicity of P2 *in vivo*, C57BL/6J-WT mice were used. 18 mice (8 weeks old) were purchased from Charles River Laboratories Spain and maintained at the Animal Facility of the Centro de Biología Molecular Severo Ochoa (CBMSO, CSIC-UAM, Madrid, Spain). After two weeks of acclimatization, mice were separated into three groups (*n* = 6) and were mock-inoculated (with PBS) or inoculated (with P2 at concentrations 5 and 50 mg/ml diluted in PBS) by inhalation for 30 min for four consecutive days. The inoculation of the animals was performed in a hermetic chamber (35 x 28 x 15 cm). The chamber has a removable perforated partition, so that it can be divided into two compartments of variable size, allowing simultaneous treatment of animals from different sex/bucket. P2 was nebulized using the portable nebulizer device OMRON CompAIR C28P (Omron Healthcare, NE-C28P), which uses air pressure to turn liquids into a mist that can be inhaled. After the exposure to the acute doses of polymer, mice were allowed free access to food and water and monitored daily for morbidity, mortality, and behavioral changes. On day 15, mice were sacrificed with CO_2_ and exsanguinated by cardiac puncture to obtain whole blood for analysis of blood counts and profiles. Several parameters were analyzed to monitor renal, hepatic, and immunological basic profiles. Body weight gain from day 0 (inoculation) to day 15 (sacrifice) was also monitored to exclude weight loss or lack of weight gain that would be indicative of toxicity.

### 2.11. *In vivo* antiviral evaluation of polymer 2

The antiviral potential of P2 was also evaluated *in vivo*. The mice used in this experiment (Tg-K18hACE2 mice) were obtained from the Jackson Laboratory [SN34860-B6.Cg-Tg (K18-hACE2) 2Prlmn/J.]. The Tg-K18hACE2 transgenic mice express hACE2 under the control of the human cytokeratin 18 promoter in airway epithelial cells (McCray et al., 2007). The original colony was expanded in our facility to produce the experimental cohort. Hemizygous animals were bred with C57BL6/J WT mice and offspring was genotyped according to Jackson's Separated PCR Assay.

**Figure 1:**
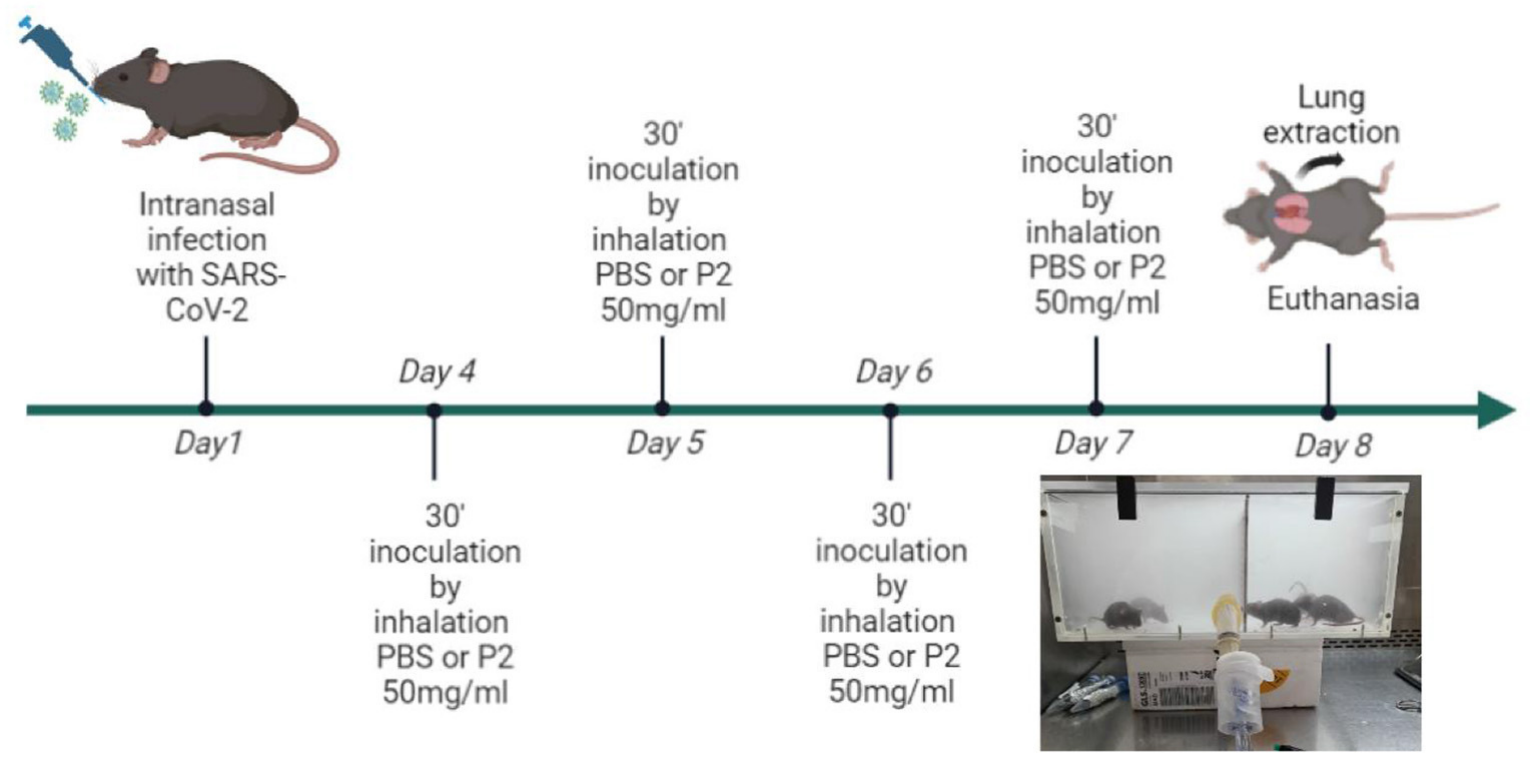
Schematic timeline of the antiviral experiments performed with SARS-CoV-2 in the mouse model C57BL6/J-k18-hACE2. The image shows the hermetic chamber where the nebulization of the animals was performed.

Tg-K18hACE2 mice (8 weeks old) were maintained in the Biosafety level 2 Animal Facility of the CBMSO. 12 Tg-K18hACE2 mice were moved to the Biosafety level 3 Animal Facility of the CBMSO, separated into two groups of six individuals each, and after acclimatization, all of them were infected intranasally with a sub-lethal dose of SARS-CoV-2 10^4^ PFU/ml of SARS-CoV-2 (Navarra 2473 strain). On days 4, 5, 6, and 7 p.i, mice were mock-inoculated (with PBS) or inoculated (with P2 at 50 mg/ml diluted in PBS) by inhalation for 30 min for four consecutive days. The inoculation of the animals was performed following the same procedure as for the toxicity test (Figure 1). Following acute dose polymer exposure, mice were allowed free access to food and water and were monitored daily for body weight, morbidity, mortality, and behavioral changes (piloerection, lethargy/stagger, eyes closed, hunched posture). Mice were euthanized at 8 d p.i, and lungs were collected from each individual. Lung tissue was processed for biomolecular analysis and histology.

### 2.12. RNA isolation

Murine lung tissues were collected in 1mL Trizol reagent (TRIReagent^®^, Sigma) and total RNA was extracted following the manufacturer's protocol. The concentration of RNA was determined by NanoDrop ND-1000 Spectrophotometer (ThermoScientific, Waltham, MA, USA) and its integrity was measured by the Bioanalyzer (Agilent 2100).

The RT-qPCR reactions were provided by the Genomics and NGS Core Facility (GENGS) at the CBMSO. The GENGS facility is part of the PTI+ Global Health (CSIC). The SARS-CoV-2 RT-qPCR assay is based on the “GENGS-3V2F SARS-CoV-2 RT-qPCR assay” [protected by CSIC as trade secret (5723-2020)], replacing the detection of the human gene for a mouse gene. Therefore, the assay detects three viral genes (N1, N2 and ORF1) and one mouse gene. This mouse gene is used as a positive internal control to confirm correct sample collection, correct RNA extraction and the absence of RT-qPCR inhibitors, to avoid false negative results. Viral genes amplifications indicate a SARS-CoV-2 RNA positive sample. Only mouse gene amplification indicates a SARS-CoV-2 RNA negative sample.

RT-qPCR reactions were performed in multiplex in 384-well plates (Shell^®^ 384-Well PCR Plates White Well Clear shell Bio-Rad CN HSP-3805) with a final volume of 10 μl and using a one-step RT-qPCR supermix. Each 10 μl reaction mixture contained 2.5 μl of mastermix, 0.2 μl of primers mix, 0.2 μl of probes and 4 μl of diluted RNA sample. To discard a potential contamination of reagents and/or primer-dimer artifacts a no template control (NTC) reaction was carried out using all the reagents except the sample. RT-qPCR reactions were performed in a CFX Opus 384 Real Time PCR System (Bio-Rad).

To determinate the number of copies of SARS-CoV-2 RNA by absolute quantification, a standard curve of Synthetic SARS-CoV-2 RNA Control 2 (Twist Bioscience, MN908947.3) was performed. This curve was obtained with a qPCR over a five-point 1/10 dilution curve made from the starting SARS-CoV-2 RNA standard concentration (10,000 copies/well). The correlation coefficient obtained for the curve was 0.993.

### 2.13. Histology and tissue staining

Half of the lung tissue was fixed in 10% formalin for 2 h and then transferred to 30 % sucrose overnight. Subsequently, the tissue was fixed with OCT and stored at −80°C. Lung sections were cut on a cryostat (LEICA CM 1950) in 15 μM slices. Sections were collected on slides (SuperFrost Plus™ Adhesion) and left at room temperature for 2–3 h and then stored at −80°C.

For hematoxylin-eosin (H&E) staining, slides were placed in the hematoxylin cuvette for 20 min (Mayer's hematoxylin solution, Sigma), once thawed at room temperature. Subsequently, they were washed with water for 5 min and briefly shaken to dry them. Then, they were stained with eosin for 2 min (5 ml 1% eosin, 180 ml 70% ethanol, 2 ml acetic acid). The slides were thoroughly washed with 100% ethanol and Neo-Clear (Sigma). Finally, slides were prepared using Neo-Mount (Sigma) as mounting medium. Images were obtained using the CKX41 Inverted Microscope and Digital Camera EP50 (Olympus-Life Science).

For immunohistochemistry analysis, the streptavidin-biotin immunohistochemical (IHC) technique (modified with the use of polymers) was used. Thermal antigen recovery was carried out by heating a pressure cooker with citrate buffer pH 6 with an electric plate, in which the slides were placed in a metal rack at maximum temperature for 3 min. After tempering, the samples were incubated in a 3% hydrogen peroxide solution in methanol and washed in TBS (tris-buffered-saline) buffer pH 7.4. The samples were then incubated in 2.5% horse serum in a humid chamber to prevent drying. Subsequently, the excess serum was removed, and the primary antibody was added: monoclonal antibody against SARS/SARS- COV2 - B46F (MA1-7404, Invitrogen). In negative controls, the primary antibody was replaced by TBS buffer. After the incubation period, the slides were washed in TBS buffer and incubated with polymer (ImmPRESS-VR Polymer Reagent, Vector Laboratories). This was followed by two washes in TBS buffer. Subsequently, development was performed (ImmPACT NovaRED Substrate Kit, Peroxidase). After developing, the samples were counterstained with haematoxylin (Gemini AS Automated Slide Stainer, Thermo Fisher Scientific) and, finally, the slides were mounted.

For lung damage semi quantification, tissue sections stained with H&E according to standard procedures for examination by light microscopy were analyzed and scored blindly for lung damage by a board-certified veterinary pathologist. A multiparametric, semiquantitative scoring system was further used to assess the magnitude of histomorphological and histopathological changes in lung tissues based on the following criteria: expansion of parenchymal wall, edema, intra-alveolar hemorrhage, inflammatory cell infiltrates, degeneration of alveolar epithelial cells, bronchiole epithelial cell damage. For each histopathological parameter, a score of 0-3 was ordinally assigned, where 0 indicated normal or no change; 1 indicated less than 10%; 2 indicated 10−50%; and 3 indicated more than 50% of lung regions affected. The cumulative scores of the severity of the three sections provided the total score per animal.

### 2.14. Ethic statements

This study was carried out in strict accordance with the European Commission legislation for the protection of animals used for scientific purposes (directives 86/609/EEC and 2010/63/EU). Mice were maintained under specific pathogen-free conditions at the CBMSO (CSIC-UAM) animal facility. The protocol for the treatment of the animals was accepted by the “Comité de Ética de la Investigación” of the Universidad Autónoma of Madrid, Spain and approved by the “Consejería General del Medio Ambiente y Ordenación del Territorio de la Comunidad de Madrid” (PROEX 168.6/22). Animals had unlimited access to food and water, and at the conclusion of the studies they were euthanized in a CO_2_ chamber, with every effort made to minimize their suffering, followed by lung collection and exsanguination by cardiac puncture to obtain whole blood.

As for human lung tissue cells, the study protocol was approved by the Clinical Research Committee [Institutional Review Board number PR(AG)212/2020] from the Vall d'Hebron University Hospital in Barcelona, Spain. Samples were obtained from adults, all of whom provided their written informed consent.

### 2.15. Statistics

All statistical analyses were performed using GraphPad Prism (version 8.0.1, GraphPad Software, Inc.). Data were subjected to Mann-Whitney-U tests (non-parametric samples) or two-tailed Student's T-tests (parametric samples) to determine significant differences between groups, and *P* values < 0.05 were considered statistically significant. For the CC_50_ and EC_50_ values, which indicate the concentration of the compound that leads to a 50% reduction in cell viability and viral infection, respectively, non-linear fit regression models were used (four parameters). For the analysis of the Kaplan-Meier survival curve, the Gehan-Breslow-Wilcoxon tests were performed.

## 3. Results

### 3.1. Antiviral assays in cell lines

#### 3.1.1. Time of addition experiments with HSV-1 K26-GFP in Vero cell line

Four different DS polymers were selected for our study: chondroitin sulfate sodium salt from shark cartilage (P1), dextran sulfate sodium salt from *Leuconostoc mesenteroides* B512F M_w_ >500,000 Da (P2), dextran sulfate sodium salt M_w_ 7,000-20,000 Da (P3), and dextran sulfate sodium salt M_r_ ~40,000 Da (P4).

**Figure 2:**
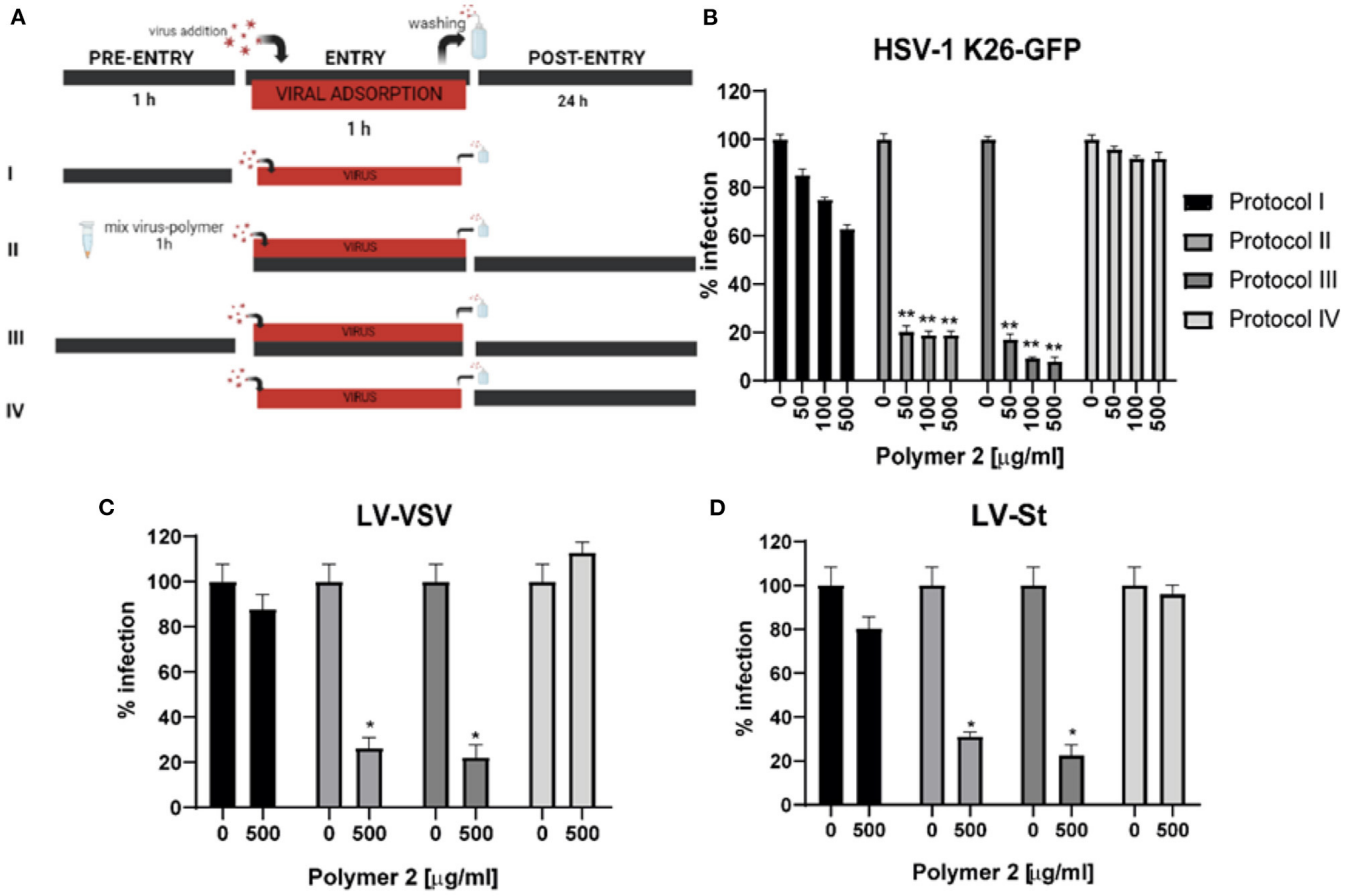
Time of addition experiments indicate that P2 interferes with early stages of HSV-1 K26 GFP, LV-VSV and LV-St infection. **(A)** Schematic figure of the times where the compound was present in the assay. Cells were cultured in 48-well plates and subjected to four different protocols**:** (I) Preincubation of cells with P2 for 1 h; (II) Mix of the virus and P2 for 1h and addition to the cells with post-incubation with P2; (III) Presence of P2 during all steps; (IV) Post-incubation of cells with P2 after viral adsorption. At 24 h, cells were collected for flow cytometry. The red bar represents viral infections; the black bar represents P2. Percentage of infection (+GFP cells) normalized to the non-infected cells for each protocol using **(B)** HSV-1 K26-GFP in Vero cells. **(C)** LV-VSV in ACE2+HEK293T cells. **(D)** LV-St in ACE2+HEK293T cells. Triplicate experiments were performed for each data point (*n* = 4), and the value is presented as mean of the percentage of normalized infection ± S.E.M. *p < 0.05 and **p < 0.0001 were considered significant as determined using the two-tailed Student's *T*-test.

Regarding time of addition experiments, HSV-1 K26-GFP was selected as the reference virus for this assay, since numerous previous studies have demonstrated the strong inhibitory activity of DSs against this virus *in vitro* (Piret et al., 2000; Witvrouw et al., 2016). Furthermore, the experiment was also assayed with pseudoviruses LV-St and LV-VSV, which enter via the interaction between the protein spike S and the cell receptor ACE2, imitating the way of entry of SARS-CoV-2. These experiments (Figure 2A) were performed in these models to confirm that the inhibitory effects of DS polymers take place on the steps of viral entry (Dyer et al., 1997; Pirrone et al., 2011). When the compounds were added before and/or during viral adsorption, they managed to reduce the viral infection (Figures 2B–D, results of P2 shown). Nonetheless, when they were added just after viral adsorption, no significant decrease in viral particles was detected. The best results were obtained when the compounds were left during all steps (Figure 2, Protocol III), where the infection drastically decreased by more than 90% in HSV-1 infection, and approximately an 80% in both pseudoviruses infections. Therefore, results suggest that the polymers interfere predominantly with the early phase of infection. We selected protocol III as the optimal one to apply in the following trials.

#### 3.1.2. Antiviral assays against enveloped viruses HSV-1, HCoV229E, LV-VSV, and LV-St

**Figure 3:**
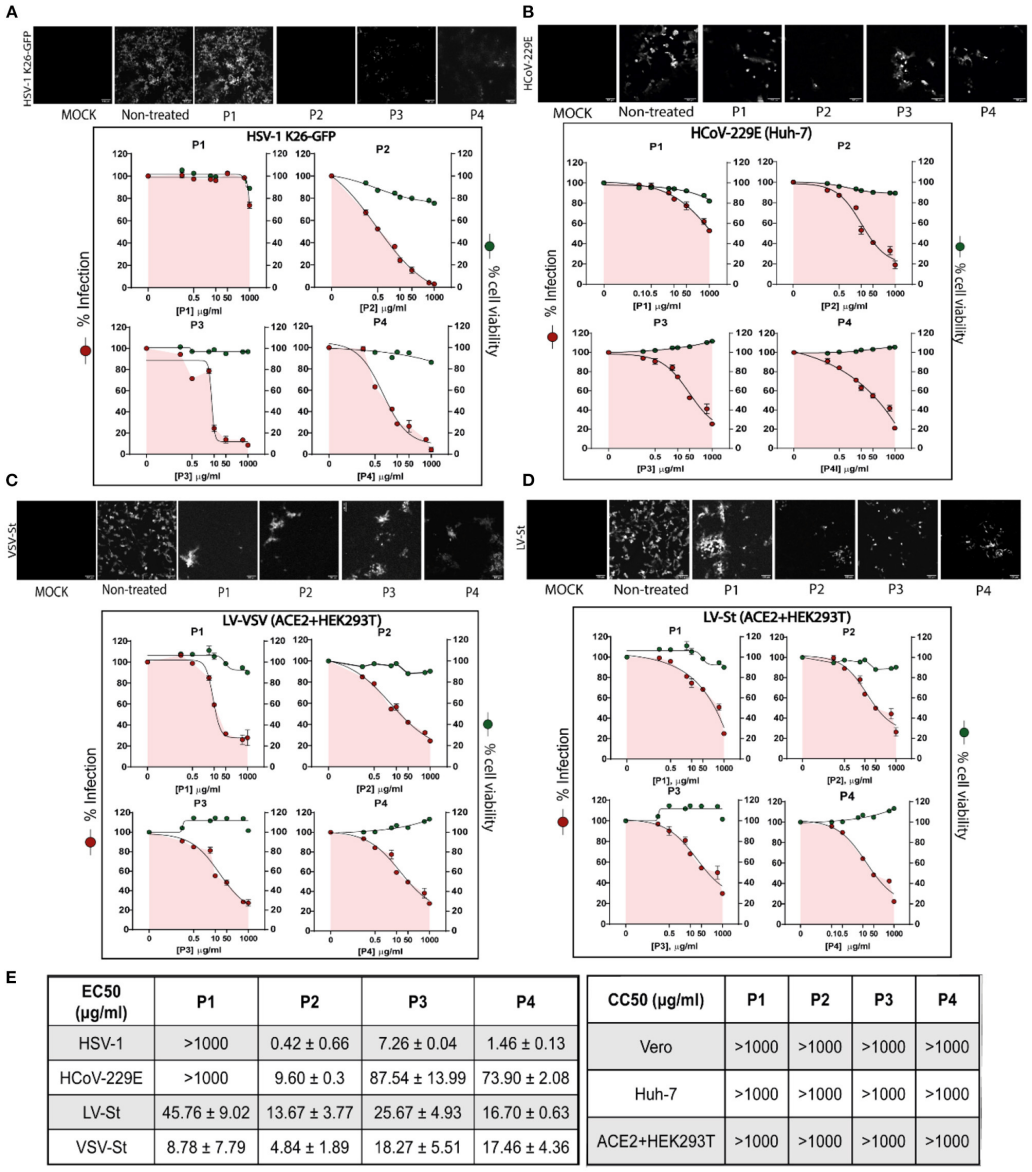
Antiviral assays and cytotoxicity of P1, P2, P3, and P4 against enveloped viruses. Results for **(A)** HSV-1 K26 GFP in Vero cell line, **(B)** HCoV-229E in Huh-7 cells, **(C)** LV-VSV in ACE2+HEK293T cells and **(D)** LV-St in ACE2+HEK293T cell line. Each cell line was incubated during all steps with the polymers at concentrations ranging from 0 to 1,000 μg/ml and subsequently infected with its corresponding virus at a specific MOI. Above, fluorescence microscopy images of cells infected with the virus show GFP+ signal corresponding to viral infection. Below, dose-response curves for EC_50_ and CC_50_ values were determined by a non-linear fit model with variable response curve (four parameters); red dots show percentage of infection and green dots represent percentage of viability compared to untreated cells. Triplicate experiments were performed for each data point (*n* = 3), and the value is presented as mean percentage of infection/viability ± S.E.M. For some conditions, S.E.M. values are so small that they are not visible on the plot. **(E)** EC_50_ and CC_50_ values ± S.D for each polymer.

First, the cytotoxicity of P1, P2, P3, and P4 at 48 h was evaluated in all cell lines by using an MTT assay. None of them exerted toxic effects at any concentration (EC_50_ values over 1 mg/ml in all cell lines tested). On the contrary, the polymers conserved cellular viability above 80%. In addition, compounds P3 and P4 were able to increase cell viability above 100% on average (Figures 3A–D).

After selecting protocol III as the optimal for the following studies (Figure 3D), different types of cell lines were cultured and infected with their corresponding viruses in the presence of the polymers at different concentrations during all steps. Infection was monitored by the expression of the GFP reporter protein, which was expressed by all viruses used in these assays. Unsurprisingly, all DS polymers (P2, P3, and P4) drastically decreased the infection at concentrations ranging from 10 to 1,000 μg/ml, but chondroitin sulfate sodium salt (P1) had no inhibitory effects on HSV1 K26-GFP infection (Figure 3A).

A similar pattern was observed for the rest of enveloped viruses tested. Fluorescence microscopy images (Figures 3A–D) reported a decrease in GFP+ signal corresponding to viral infection in cells treated with the compounds compared to untreated samples, a decrease that was lower in P1-treated cells. Except for the VSV-St virus, P1 reported the highest EC_50_ values (Figure 3E). In addition, P2 and P4 were the compounds that reported the lowest EC_50_ values in the non-linear fitting regression curves against HSV 1 K26-GFP, HCoV-229E, LV-VSV, and LV-St infections, with a wide protective window in all tested cell lines.

All polymers neutralized the entry of the SARS-CoV-2 S protein-pseudotyped lentivirus LV-St in a dose-dependent manner. This, added to the time-of-addition experiments, suggests that the candidate compounds primarily interfere with early aspects of infection.

#### 3.1.3. Antiviral assays against SARS-CoV-2

**Figure 4:**
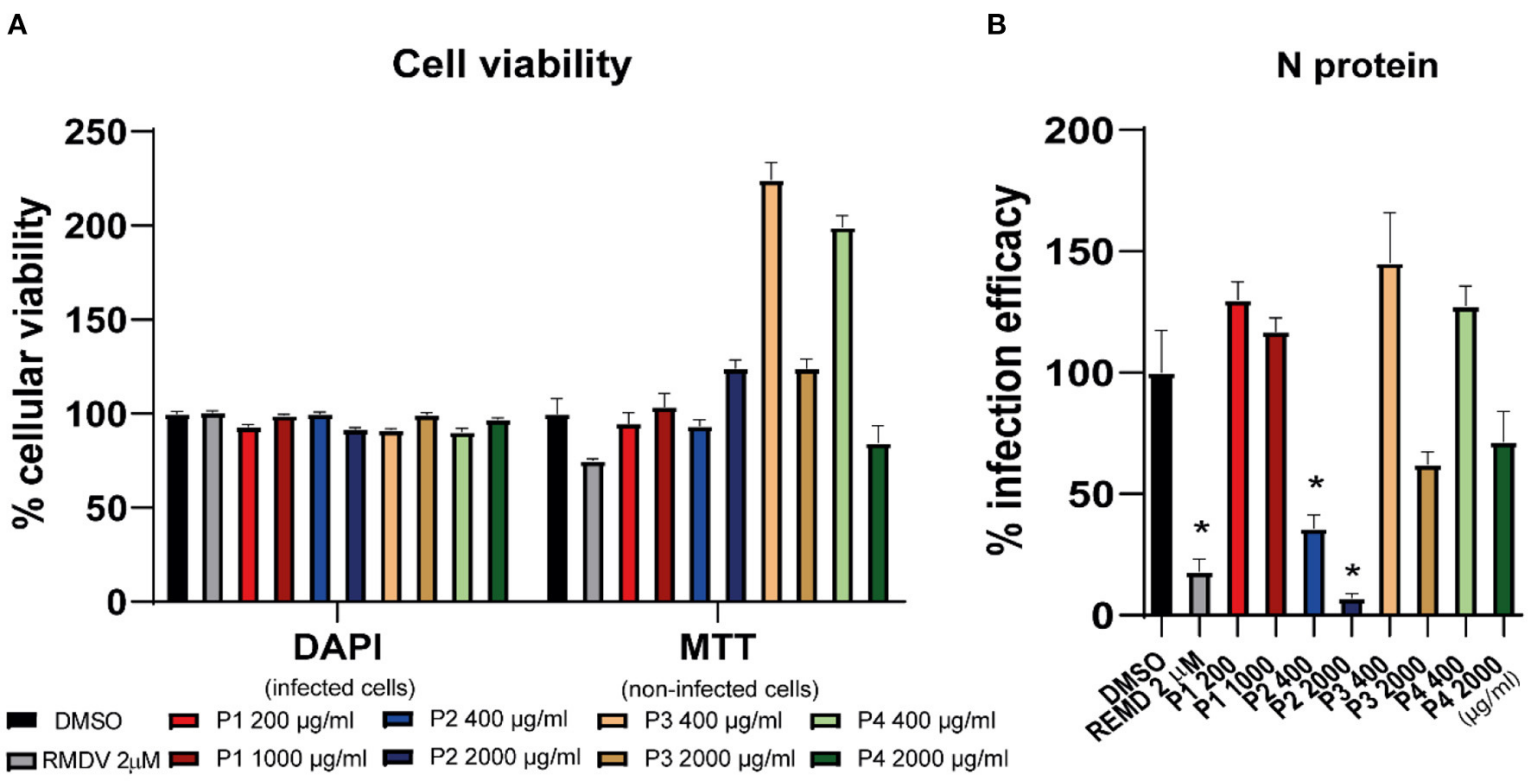
P2 decreases the infection of SARS-CoV-2 in Vero-E6 cells *in vitro* at non-cytotoxic concentrations. Vero-E6 cells were inoculated at a MOI of 0.01 with SARS-CoV-2 in the presence of DMSO (vehicle), remdesivir (positive control), and the four polymers. At 48 h post-infection (p.i.), cells were fixed with PFA 4% PBS and processed for immunofluorescence microscopy. Nuclei of infected samples were stained with DAPI. Uninfected cultures were also tested for viability in the presence of the same compound doses using an MTT-based assay. **(A)** Percentage of viability of infected cells (DAPI) and non-infected cells (MTT) in the presence of the compounds for 24 h. Results were normalized to the DMSO-treated cells and are shown as mean ± S.D (*n* = 3). **(B)** Infection efficiency measured as N protein expression reduction and expressed as the percentage of that observed in vehicle DMSO-treated cells and is shown as mean ± S.E.M. (*n* = 3). *p < 0.01 was considered significant as determined using the Mann-Whitney-U test.

To confirm the antiviral potential of the candidate polymers against SARS-CoV-2, they were diluted and mixed with a virus stock to inoculate Vero E6 cells. The antiviral activity was further confirmed by immunofluorescence microscopy, to estimate virus propagation. Cell viability was also evaluated in parallel to infection (DAPI, Figure 4A) and in non-infected cells (MTT, Figure 4A). No cytotoxicity was observed at the assayed doses. In fact, compounds P3 and P4 showed again high viability values in the MTT assay. As for the antiviral assay, DS from *L. mesenteroides* (P2) was highly effective in reducing infection efficiency, as suggested by SARS-CoV-2 N protein staining (Figure 4B). Furthermore, P2 reduced the infection to the same levels as the cells treated with RMDV (positive control), the only clinically approved antiviral for the treatment of COVID-19 patients (National Insitutes of Health Antiviral Therapy, 2022).

#### 3.1.4. Antiviral assays against the non-enveloped minute virus of mice

**Figure 5:**
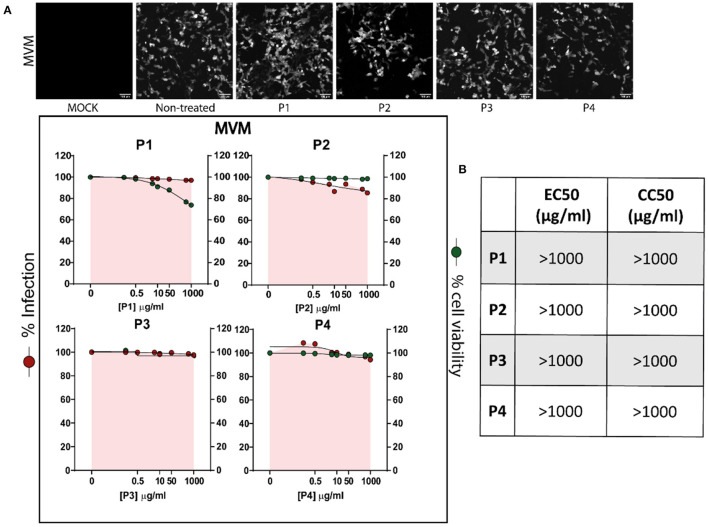
Polymers P1, P2, P3, and P4 do not exert antiviral activity against non-enveloped virus MVM in the HeLa cell line at non-toxic concentrations. Cells were incubated all the time with the polymers at concentrations ranging from 0 to 1,000 μg/ml, and subsequently infected with MVM at a MOI of 0.5. **(A)** Above, fluorescence microscopy images of cells infected with the virus show GFP+ signal corresponding to viral infection. Below, dose-response curves for EC_50_ and CC_50_ values were determined by a non-linear fit model with variable response curve (four parameters); red dots show percentage of infection and green dots represent percentage of viability compared to untreated cells. Triplicate experiments were performed for each data point (*n* = 3), and the value is presented as mean of the percentage of normalized infection ± S.E.M. For some conditions, S.E.M. values are so small that they are not visible on the plot. **(B)** EC_50_ and CC_50_ values for each polymer in HeLa cells.

To assess whether the antiviral activity of the DS polymers was limited to enveloped viruses (Bello-Morales et al., 2022), the compounds were tested against non-enveloped MVM in the HeLa cell line. As expected, none of the polymers were able to reduce the infection of this virus (Figure 5A). Furthermore, none of the compounds exhibited cytotoxic effects (CC_50_ values greater than 1,000 μg/ml) (Figure 5B), but whereas fluorescence microscopy images and flow cytometry data revealed that the percentage of infection remained around 100%.

### 3.2. Antiviral assays in human lung tissue cells

Following the promising results obtained in cell lines models, the next step was to demonstrate the antiviral activity of DS polymers in models closer to the clinic. A rapid platform for the identification of viral entry inhibitors by using human lung tissue (HLT) was used (Grau-Expósito et al., 2022). Cell suspensions from primary HLTs were obtained from three different patients with negative PCR tests for SARS-CoV-2, processed, and cultured for the assay. HLT cells were subjected to VSV^*^ΔG (Luc)-Spike virus in the presence of a 1/5 serial dilution of the different candidates tested. 20 h post-exposure, antiviral activity and cell viability were measured by luminescence. Camostat mesylate was the drug used as a positive control (results not shown) due to previous reports describing high antiviral activity in this HLT model (Grau-Expósito et al., 2022) and in precision-cut lung slices (Hoffmann et al., 2021).

**Figure 6:**
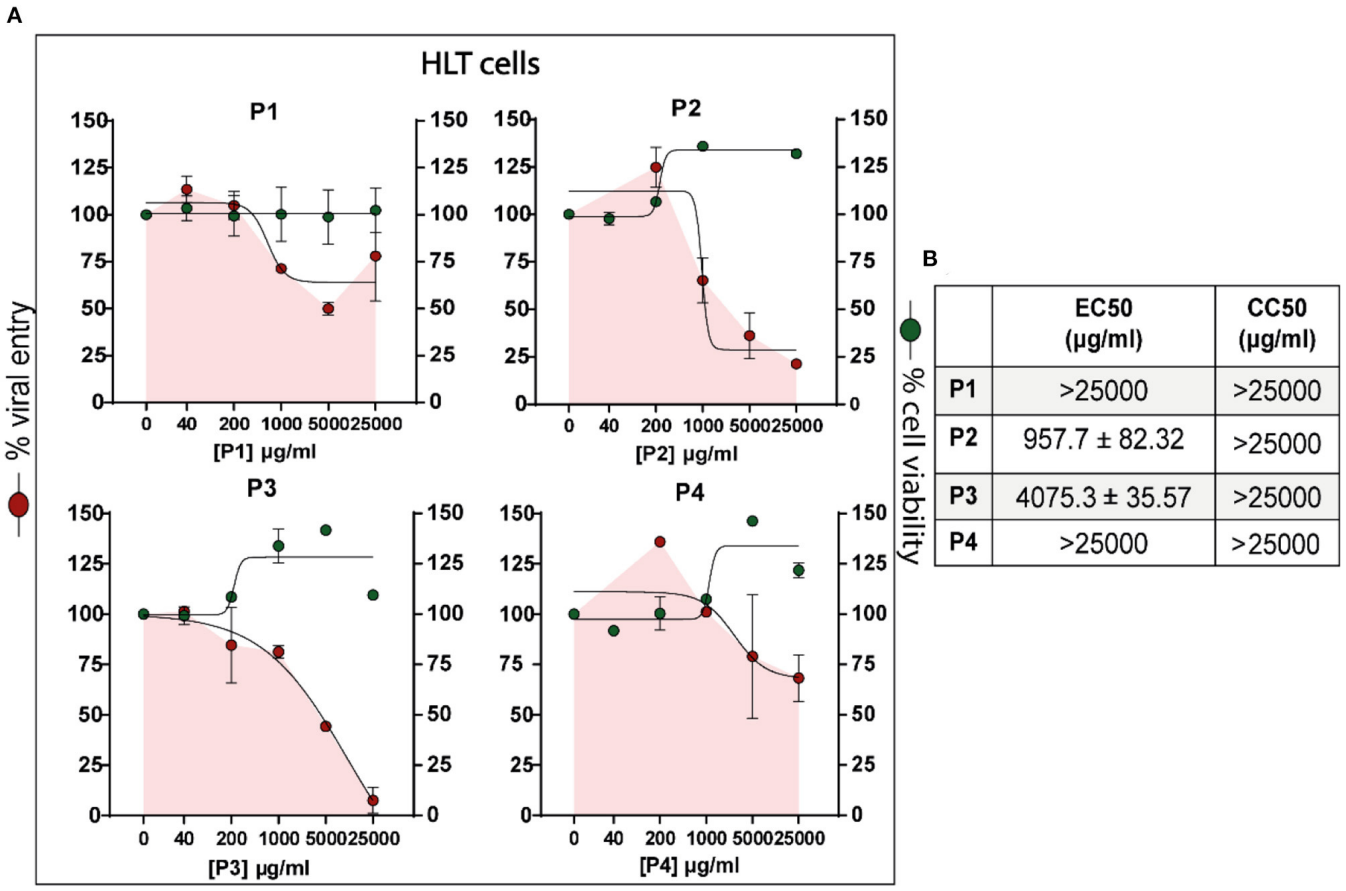
Percentage of viral entry in HLT cells exposed to VSV*ΔG (Luc)-Spike in the presence of compounds P1, P2, P3, and P4. HLT cells were incubated with VSV*ΔG (Luc)-Spike virus in the presence of a 1/5 serial dilution of the different polymers tested. Antiviral activity and cell viability were measured 20 h p.i by luminescence. **(A)** Non-linear fit model with variable response curve (four parameters) from at least three independent experiments in replicates is shown (red lines). Cytotoxic effect on HLT exposed to drug concentrations in the absence of virus is also shown (green lines). **(B)** CC_50_ and EC_50_ values of each drug for HLT cells. Triplicate experiments were performed for each data point (*n* = 3), and the value is presented as mean of the percentage of viral entry/viability ± S.E.M.

Preliminary assays revealed that the calculated EC_50_ and CC_50_ values in cell line models differed drastically from the values in HLT cells for the same concentration. Therefore, the concentration of polymers was increased, with 25 mg/ml being the maximum concentration tested. Polymers P2 and P3 were the compounds that most effectively inhibited SARS-CoV-2 entry into HLT cells without affecting cell viability (EC_50_ values of 972.7 and 4275.3 μg/ml, respectively). Therefore, the potential benefit of DS from *L. mesenteroides* during the early phase of infection was again demonstrated. Nonetheless, P1 and P4 induced a slight viral entry suppression at high concentrations (Figures 6A, B).

### 3.3. *In vivo* toxicity evaluation of polymer 2 in mice

After confirming the tolerability and antiviral efficacy of the polymers *in vitro*, with DS from *L. mesenteroides* (P2) standing out as the compound that achieved the lowest EC_50_ values in immortalized cell lines and primary cell cultures, we wanted to evaluate whether inhalation treatment could be effective in protecting mice susceptible to SARS-CoV-2 infection.

**Figure 7:**
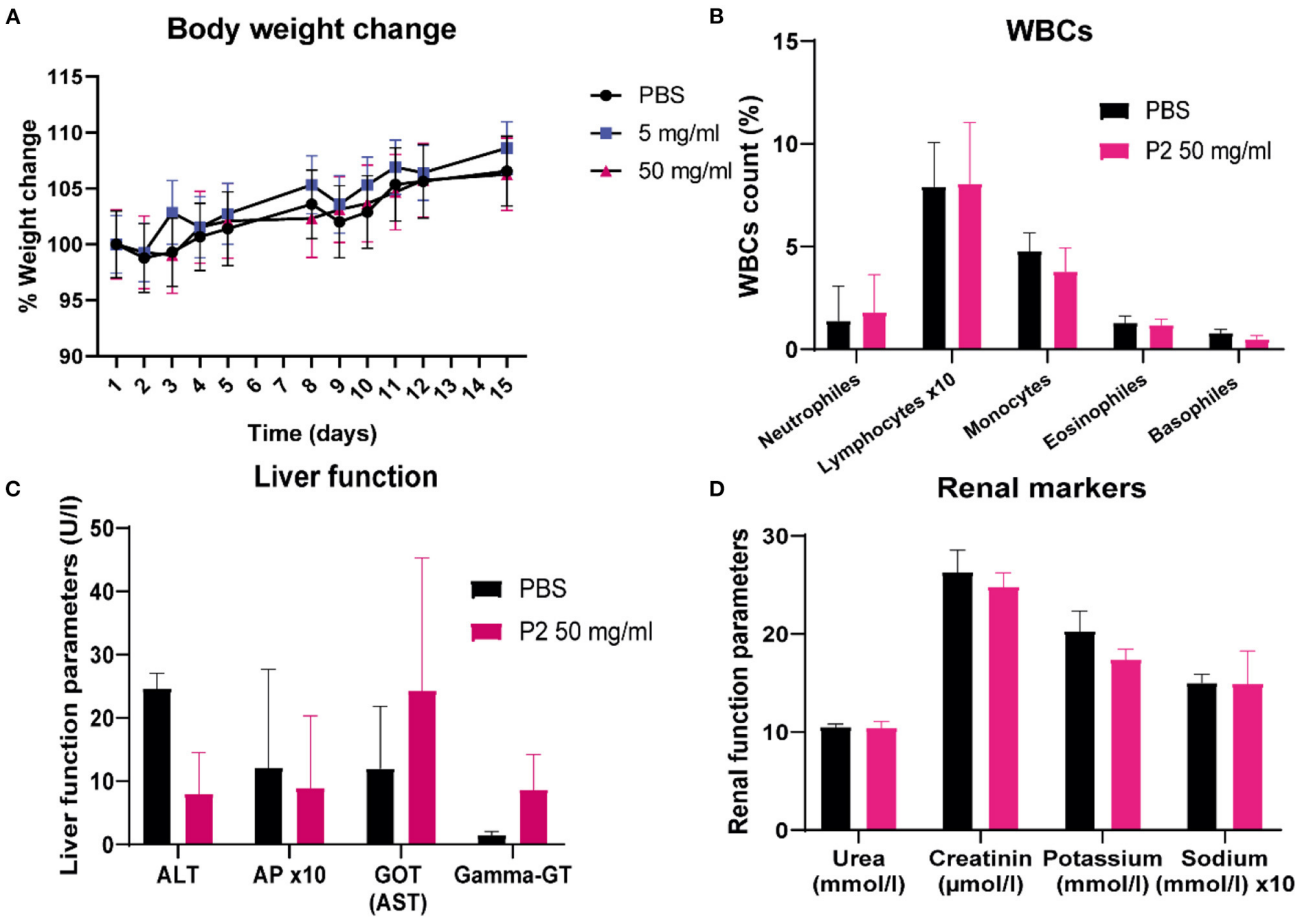
Polymer 2 shows no toxic effects in an *in vivo* mouse model. Eighteen C57BL6/J WT mice were distributed in three different groups (*n* = 6) and were mock-inoculated (PBS) or inoculated (P2 at the concentrations of 5 or 50 mg/ml diluted in PBS) by inhalation for 30 min for four consecutive days in a hermetic chamber. On day 15, mice were sacrificed and whole blood was obtained by cardiac puncture. **(A)** Percentage of body weight change, **(B)** WBC count, **(C)** liver markers alanine transaminase (ALT), alkaline phosphatase (AP), aspartate aminotransferase (AST), and Gamma-glutamyl transferase (Gamma-GT), and **(D)** renal markers urea, creatinine, potassium, and sodium levels were analyzed. Mean (*n* = 6) ± S.E.M are shown, and statistical comparisons were performed using the Mann-Whitney-U test.

First, the toxicity evaluation of the candidate compound was performed. 18 C57BL6/J mice were mock-inoculated or inoculated with different single doses of P2 for four consecutive days (Section 2.10). Several parameters were analyzed, such as body weight change (Figure 7A). Regarding WBCs count (Figure 7B), liver (Figure 7C), and renal markers (Figure 7D), all data remained within normal parameters established in mice by the clinical laboratory responsible for the biochemical analysis. ALT and AP enzymes in P2-treated mice seemed to decrease, while GOT and Gamma-GT values tended to increase, but without statistical significance. For 15 days there were no significant changes in any of the parameters between the control and experimental groups. No toxic signs such as hypothermia, weakness, diarrhea, or ataxia were observed. Hypochromia and anisocytosis tests were negative. There were also no signs of acute pain, distress, or weight loss. The maximum concentration tested (P2 50 mg/ml) did not show any of these signs and was selected as the dose used for the antiviral assay.

### 3.4. *In vivo* antiviral activity evaluation of P2 in mice

**Figure 8:**
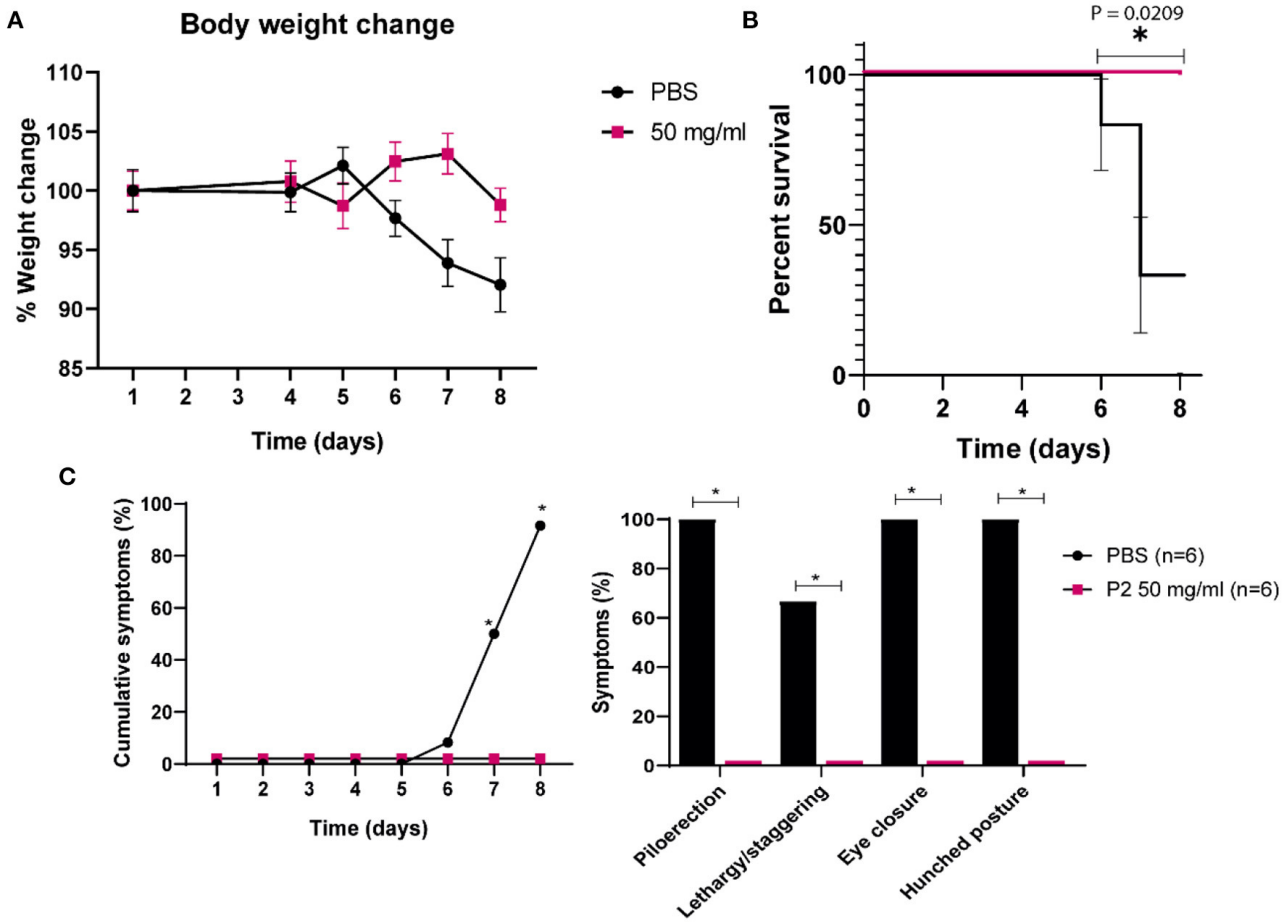
Percent survival and clinical signs of Tg-K18hACE2 mice infected with SARS-CoV-2 and treated with P2 by inhalation. Twelve Tg-K18hACE2 mice were distributed into two groups of six individuals each and infected with 10^4^ PFU/ml of SARS-CoV-2 (Navarra strain 2473) intranasally. On days 4, 5, 6, and 7 p.i, mice were mock-inoculated (PBS) or inoculated (50 mg/ml P2) by inhalation for 30 min in a hermetic chamber. The mice were euthanized on day 8 p.i. Changes in body weight **(A)**, percent survival **(B)**, and clinical signs **(C)** were monitored daily. The analyzed symptoms (excluding weight loss) consisted of piloerection, lethargy and staggering, eye closure, and hunched posture. Mock-infected mice did not exhibit any symptoms throughout the experiment. The number of cumulative symptoms, exhibiting at least one of the previously described, are represented for each group. The individual symptoms are considered as positive when a mouse showed it at any day during the period 7–8 dpi. Mean (*n* = 6) ± S.E.M. are shown, and statistical comparisons were performed using the Gehan-Breslow-Wilcoxon test; *p < 0.05.

Being 50 mg/ml the concentration of P2 that exhibited no toxic effects in mice, its antiviral effect against SARS-CoV-2 was tested *in vivo*. Tg-K18hACE2 mice were infected intranasally with 10^4^ PFU/ml of SARS-CoV-2, and treated or mock-treated on days 4, 5, 6 and 7 with nebulized P2 50 mg/ml, as previously described. Animals treated with P2 gained weight (Figure 8A), while those treated with PBS started to stop eating and thus their body weight decreased. Kaplan-Meier survival curve (Figure 8B) reports that 50% of the PBS-treated mice died before the end of the experiment, showing a significant mortality kinetic. In contrast, none of the P2-treated individuals died prematurely. Regarding clinical signs (Figure 8C), all PBS-treated mice exhibited piloerection, lethargy, eye closure, and hunched posture on days 7–8 p.i. These results report a significant difference in morbidity, since mice treated with P2 remained clearly asymptomatic.

**Figure 9:**
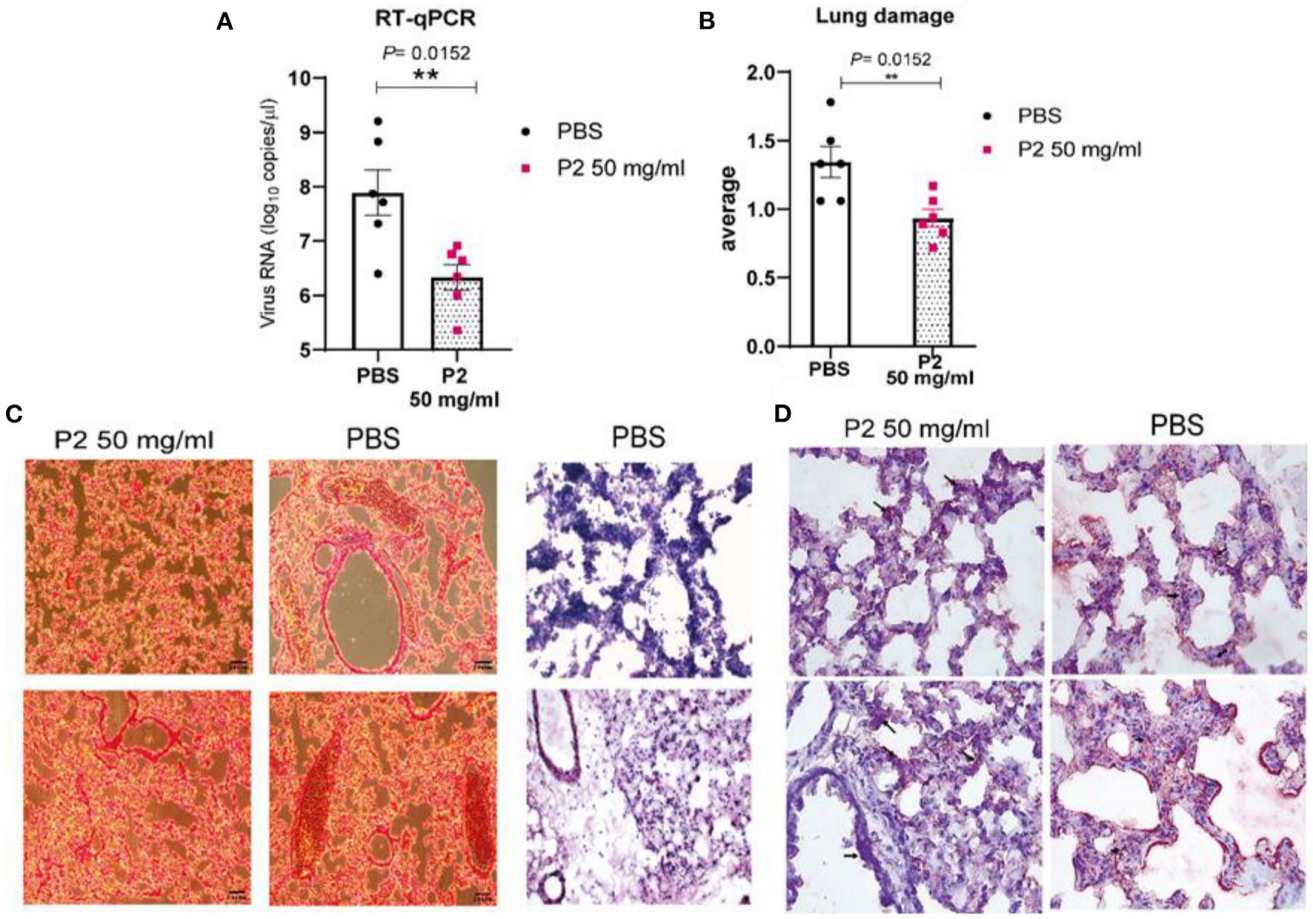
Effect of P2 on viral RNA levels and histology of Tg-K18hACE2 mice lung samples. **(A)** Viral genome copies in lung tissue determined by RT-qPCR. **(B)** Semi-quantification of the lung damage. **(C)** Hematoxylin and eosin (H&E) staining and **(D)** immunohistochemistry (IHC) images of N protein of SARS-CoV-2 of infected mice lung tissue 8 days p.i (scale bar, 100 μm, *n* = 6), treated with either PBS or 50 mg/ml P2. Arrows show the presence of cumulative virions. Two representative cryosections from two different mice of the same group are shown. Mean (*n* = 6) ± S.E.M. are shown. Statistical comparisons were made using Mann-Whitney-U tests; **p = 0.0152.

The viral load in the lungs of the mice was also calculated by quantitative reverse transcription PCR (RT-qPCR). Virus yield in lung tissues was significantly reduced in P2-treated mice, suggesting that viral replication has been suppressed (Figure 9A).

Finally, to evaluate whether treatment with P2 at a concentration of 50 mg/ml caused any significant morphological difference in lung tissue compared to PBS-treated mice, histopathological studies were performed. To assess this, lung tissue was analyzed by histology on day 8 p.i. Lung damage quantification reveals significant differences between treated and non-treated animals (Figure 9B). This damage was evaluated by detecting an expansion of the parenchymal wall, desquamation and degeneration of alveolar epithelial cells, edema, and multi-nucleated cell formation, among others. Gross pathology revealed macroscopic manifestations of red lesions and discoloration in lungs of PBS treated mice (Figure 9C, Supplementary Figure 2). In the PBS treatment group, extensive lung epithelial surface disruption and cellular debris are observed; however, no such disruptions were found in the 50 mg/ml P2 group. Such features of diffuse alveolar damage have been described in human lung tissues of patients with positive PCRs for SARS-CoV-2 and COVID-19 symptoms. To support RT-qPCR results, SARS-CoV-2 N protein expression was detected in lung tissue from infected mice by immunohistochemistry (IHC) (Figure 9D). In PBS-treated mice, the staining corresponding to the presence of the virus was more intense compared to the P2-treated ones. These results indicate that P2 is well tolerated by the lung epithelium both *in vitro* and *in vivo* and that this polymer exerts promising antiviral efficacy at the 50 mg/ml concentration *in vivo*.

## 4. Discussion

Dextrans are high molecular weight branched glucopolysaccharides produced from sucrose by lactic acid bacteria (LAB) belonging to the *Lactobacillaceae* family. The DS P2 used in this work is a dextran derived from *Leuconostoc mesenteroides* strain B512F. It is composed of a linear chain of glucose monomers with approximately 95% α-D-(1,6) linkages, accounting the remaining α-D-(1,3) linkages for the branching of dextran. Regarding the branch lengths, the average branch length is less than three glucose units (Lindberg and Svensson, 1968; Larm et al., 1971), although other methods have indicated branches of greater than 50 glucose units (Senti et al., 1955; Bovey, 1959).

The safety of dextrans is endorsed by the inclusion of dextran 70 in the 22nd (2021) WHO Model List of Essential Medicines [World Health Organization (WHO), 2021]. On the other hand, the toxicity of dextran sulfate sodium (DSS) depends on its molecular weight. DS orally administered to humans did not exert significant side effects or systemic absorption (Abrams et al., 1989). However, administration of 1–5% high molecular weight DSS in drinking water induced acute intestinal injury in mice (Chassaing et al., 2014; Kiesler et al., 2015; Munyaka et al., 2016; Park et al., 2021).

DS is anticoagulant when administered intravenously. When administered by infusion, this polymer has shown to trigger, among other side effects, mild epistaxis, thrombocytopenia or transient elevations in alanine and aspartate aminotransferases (Flexner et al., 1991). However, the effects of DS administered by inhalation in humans or animal models had not been reported before. Here we have demonstrated for the first time the absence of significant side effects of DS after inhalation administration in a mouse model.

Numerous studies have also correlated the molecular weight and degree of sulfation of sulfated polysaccharides with their antiviral activity (Ray et al., 2022). Our *in vitro* assays support this hypothesis since P2 (M_w_: 500kDa) reported the lowest EC_50_ values, followed by P4 (M_w_: 40 kDa) and finally P3 (M_w_: 7–20 kDa) (Witvrouw et al., 2016). However, P1 (chondroitin sulfate from shark cartilage) did not show remarkable antiviral effects. Chondroitin sulfate polymers have been shown to have weaker antiviral effects compared to DS polymers in HSV-1 (Nyberg et al., 2004), and only the chondroitin sulfate type E purified chain from squid cartilage has exhibited potent antiviral activity against HSV-1 (Bergefall et al., 2005). As for SARS-CoV-2, chondroitin sulfates do not show competitive binding to S protein (Kwon et al., 2020), and a recent study reported no activity of chondroitin sulfate against VSV- pseudotyped SARS-CoV-2 vector (Izumida et al., 2022).

The mechanism of action by which DSs exert their antiviral activity depends mainly on non-covalent interactions between the negative charges of the polymers and the positive charges on the virion envelopes (Bello-Morales et al., 2022). This interaction is not specific, as DS polymers have demonstrated antiviral activity against several viruses that use different receptors to enter cells. Furthermore, the polymers tested in our study lack inhibitory activity against the non-enveloped virus MVM, supporting the key role of the interaction between the viral envelope and the compounds. This suggests that their antiviral properties might be a universal phenomenon against enveloped viruses. Not only electrostatic forces, but also van der Waals forces, H-bonds, hydrophobic effects, cation bridging, or steric interactions favor the contact of virions and DSs (Bello-Morales et al., 2022).

**Figure 10:**
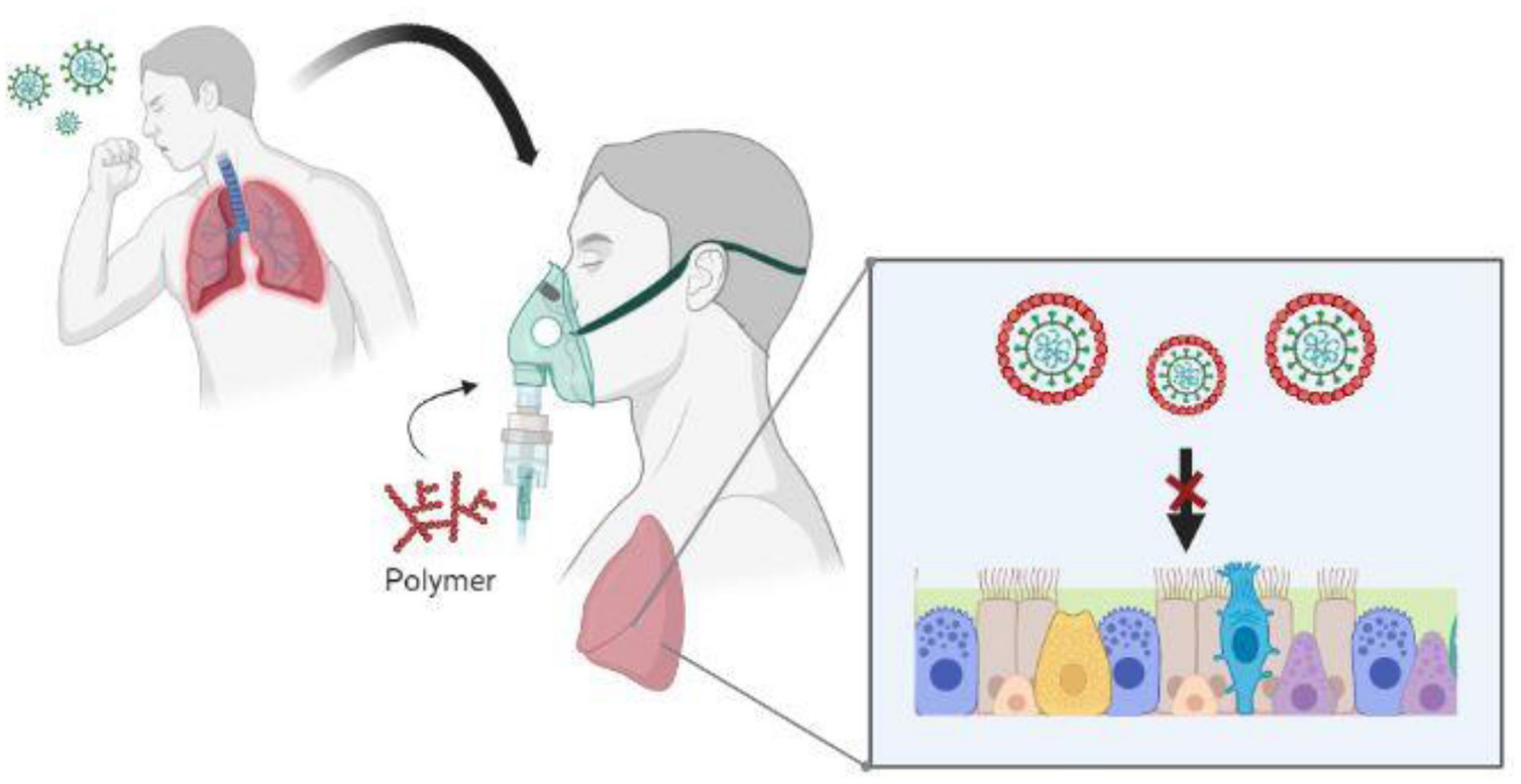
Possible strategy of administration of P2. In a patient with suspected SARS-CoV-2 infection, or in early stages of infection, the DS-based polymer is nebulized via aerosols to access the nasal cavity and pulmonary alveoli. Airborne virions can be trapped by the polymer before they attach and enter the respiratory epithelium, thus reducing viral infection.

Since these polymers have shown low toxicity and potent antiviral activity *in vitro* and *in vivo*, we suggest that they may have promising preventive clinical use, also due to their low cost and ease of production. SARS-CoV-2 infection begins via respiratory droplets that are deposited in the nasal, conjunctival, and oral mucosa. Receptors for SARS-CoV-2 are mainly expressed in epithelial cells in the nasal cavity (goblet cells and a subset of ciliated cells), and type II pneumocytes in alveoli (Emrani et al., 2021). Time of addition experiments and testing in HLT cells have demonstrated that the effect of our polymers takes place mainly in the early steps of the infection. Therefore, we propose the strategy of directly attacking the virus just when it reaches the upper respiratory tract, the lungs, and other target cells present in the airways. This timing is ideal for polysulfates to trap airborne coronaviruses at the respiratory tract level, based on the physical chemistry of polyelectrolyte complexes (Nie et al., 2021; Vert, 2021). Nonetheless, viral replication and shedding could continue for several weeks in the case of severe patients, so repetitive administration should be considered. As part of post COVID-19 syndrome, the persistence of respiratory symptoms, especially dyspnea and cough, beyond four weeks from the outbreak of symptoms appears to be common (Vadász et al., 2020; Montani et al., 2022). Therefore, the precise treatment of respiratory symptoms is important in long-term prospective follow-up studies. The window to treat emerging SARS-CoV-2 infection before its peak is longer in humans compared to the mouse model (Sheahan et al., 2020), and our trials were performed in young mice (disease severity increases with age), so this must be taken into account in treatment design. The main drawback of polyanions is their low bioavailability, which can be avoided by using appropriate administration strategies here proposed, such as gargling, inhalation, and nasal spraying of an aqueous solution to access the oral and nasal cavities, or aerosol nebulization (Figure 10) to access pulmonary alveoli (Bello-Morales et al., 2022). All things considered, we suggest the inhalation with nebulized P2 in combination or not with current antiviral therapies for prevention.

In conclusion, this study highlights the broad-spectrum antiviral properties of exopolymers produced by *L. mesenteroides* B512F against SARS-CoV-2, and it also shows that inhalation is a suitable administration route for treating the infection. *In vivo* assays reported in this work showed no signs of toxicity and demonstrated a drastic inhibition of SARS-CoV-2 infection in mice treated with dextran sulfate 50 mg/ml. In addition, it is broadly active *in vitro* against various enveloped viruses, including the coronavirus HCoV-229E, and HSV-1. The low cost, speed of production, and the ease of application makes this polymer a good alternative for the prevention of COVID-19.

## Data availability statement

The data that support the findings of this study are available from the corresponding author, SA, upon reasonable request. Some data are not available due to ethical requirements.

## Ethics statement

As for human lung tissue cells, the study protocol was approved by the Clinical Research Committee [Institutional Review Board number PR(AG)212/2020] from the Vall d'Hebron University Hospital in Barcelona, Spain. Samples were obtained from adults, all of whom provided their written informed consent. The patients/participants provided their written informed consent to participate in this study. This study was carried out in strict accordance with the European Commission legislation for the protection of animals used for scientific purposes (directives 86/609/EEC and 2010/63/EU). The protocol for the treatment of the animals was accepted by the Comité de Ética de la Investigación of the Universidad Autónoma of Madrid, Spain and approved by the Consejería General del Medio Ambiente y Ordenación del Territorio de la Comunidad de Madrid (PROEX 168.6/22).

## Author contributions

Conceptualization: SA, RB-M, and JL-G. Methodology: SA, RB-M, CK, PD, MB, NG, JM-S, MG, and GR. Formal analysis, investigation, and writing—original draft preparation: SA and RB-M. Writing—review and editing: SA, RB-M, IR, JL-G, PD, SZ, LE, NG, and CK. Supervision: RB-M, JL-G, PD, NG, JM-S, CK, and MB. Funding acquisition: JL-G. All authors have read and agreed to the published version of the manuscript.
