# Supplementary material for: Dextran sulfate from Leuconostoc mesenteroides B512F exerts potent antiviral activity against SARS-CoV-2 in vitro and in vivo
Source: Front Microbiol. 2023 May 3;14:1185504. doi: 10.3389/fmicb.2023.1185504 (PMC10189130; doi:10.3389/fmicb.2023.1185504)
Supplement: Supplementary file 1 [file Data_Sheet_1.docx]

## Certificate of Origin

**A**

**Product Number:** C4384

Outside USA [eurtechserv@sial.com](mailto:eurtechserv@sial.com)

#### **Product Name:** Chondroitin sulfate sodium salt shark cartilage sulfated glycosaminoglycan polysaccharide

**S1**

**Lot Number:** BCCC9552

**Brand:**

Country of Manufacture: China

Type of Manufacture

Chemical synthesis / chemical transformation Fermentation / from cell culture Purification/extraction from biological material

Purification/extraction from non-biological natural inorganic/organic source

Is the product / material partly or fully of human or animal origin (i.e., tallow derived materials, tissue, tissue extract or fluid such as milk, blood, etc.)?

Does the manufacturing process involve any raw materials, sourcing materials, or reagents that are of human or animal origin (i.e., lubricants, cleaning agents, filtering agents, processing aids, enzymes or cultural media, fermentation)?

Is any of the equipment used for processing and / or storage of the product in contact at any time with other materials of human or animal origin (i.e., cleaning solutions, solutions for equipment, test media used in equipment validation, other human or animal-derived products / materials)?

Is the product / material, for any other reason, at any time during its manufacturing process, in contact with materials of human or animal origin including fermentation?

Does the closure container (e.g. bottles, caps, drum, etc.) involve any raw materials that are of human or animal origin?

No No Yes No

Yes

Yes

No

No

No

Is the Product/Material synthetic origin? No

Are there procedures in place to avoid cross contamination with other materials or residue of animal, human, GMO origin or Allergen materials?

Is the product/material directly produced through genetic engineering?

**Yes**

No

Genetically modified organisms used No

Have any gluten materials been applied during No manufacturing?

Solvent(s) are used Yes

- Ethanol <= 5000 ppm

During the manufacturing process is a catalyst(s) used? No

Is the product/material directly produced or handled using No ionizing radiation?

During the manufacturing process is a solvent(s) used? Yes

Are processes in-place for monitoring the production process, traceability and batch consistency?

Is this product/material collected and/or processed in dedicated equipment?

Yes No

Outside USA [eurtechserv@sial.com](mailto:eurtechserv@sial.com)

Was this product/material collected and packaged in new or sterilized containers?

**Yes**

Tap Water Yes

Steam Yes

Spray Yes

Have any allergen materials been applied during manufacturing?

**Yes**

Biological Origin Animal

Species of Animal

- Fish

Country of Origin of Animal CN, ID

Has a Certificate of Suitability been filed with the EDQM for No TSE (Transmissible Spongiform Encephalopathies) on your

product?

Are the animal species traceable back to the location of No birth?

Was animal protein fed to the animal(s)? No

Was ruminant protein fed to the animal(s)? No

Animal Part Used: Shark cartilage

Were animals slaughtered or did tissue come from donor animals.

slaughter

Did animal(s) pass ante and post mortem inspection? Yes

Does any of this material contain or been in contact with No neuronal tissue?

Was collected tissue commingled in any way with other No animal material?

Was tissue collected post mortem? Yes Was the collected tissue deemed fit for human consumption? Yes

Has the processing site been inspected by the USDA, EU or No equivalent government agency?

**Document issued by Sigma-Aldrich Corporation "Sigma-Aldrich". This document is valid without signature and has been produced digitally.**

**This information is to be used for the purpose of determining animal or other biological origin only and not to be confused with "country of origin" for import/export purposes. Data provided in this document are property of Sigma-Aldrich.**

**The above information is believed to be correct to the best of our knowledge**

**Sigma-Aldrich shall not be held liable for any damage resulting from the sale, use, handling or from processing the above product(s). This document does not make any warranty, express or implied, of fitness for any particular use of the product(s).**

**Purchaser must determine the suitability of the product(s) for its use under the applicabble laws and regulations.**


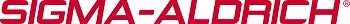

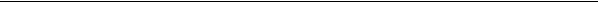


# Certificate of Analysis

3050 Spruce Street, Saint Louis, MO 63103 USA

Email USA: [techserv@sial.com](mailto:techserv@sial.com) Outside USA: [eurtechserv@sial.com](mailto:eurtechserv@sial.com)

#### **Product Name:** Chondroitin sulfate sodium salt shark cartilage sulfated glycosaminoglycan polysaccharide

**Product Number:** C4384

**Batch Number:** BCCC9552

**Brand:** Sigma

**CAS Number:** 9082-07-9

**Formula:**

**Formula Weight:**

### Storage Temperature: 2-8 C

**Quality Release Date:** 20 APR 2020

**Recommended Retest Date:** FEB 2022

### TEST SPECIFICATION RESULT

**APPEARANCE (COLOR)** WHITE TO OFF WHITE OFF-WHITE

**APPEARANCE (FORM)** POWDER POWDER

**REMARKS ON HPLC** 6-SULFATE:4-SULFATE RATIO

≥0.33:1

6-SULFATE:4-SULFATE RATIO 1.64:1

**SOLUBILITY (COLOR)** COLORLESS TO VERY FAINT YELLOW ALMOST COLORLESS

**SOLUBILITY (TURBIDITY)** CLEAR TO SLIGHTLY HAZY CLEAR

**SOLUBILITY (METHOD)** 10MG/ML IN 0.9% SODIUM CHLORIDE 10MG/ML IN 0.9% SODIUM CHLORIDE

**WATER** ≥1 % 4.7 %

**ASSAY (ICP)** SODIUM ≥4 %; SULFUR ≥ 4 % SODIUM 6.9 %; SULFUR 5.9 %

**RESIDUAL SOLVENTS (GLC-HS)** ≤4 % 1.6 % ETHANOL


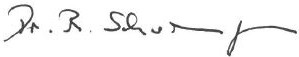


Dr. Reinhold Schwenninger Quality Assurance

Buchs, Switzerland

Sigma-Aldrich warrants that at the time of the quality release or subsequent retest date this product conformed to the information contained in this publication. The current specification sheet may be available at Sigma-Aldrich.com. For further inquiries, please contact Technical Service. Purchaser must determine the suitability of the product

for its particular use. See reverse side of invoice or packing slip for additional terms and conditions of sale.


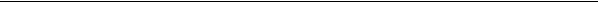


Sigma-Aldrich Certificate of Analysis - Product C4384 Lot BCCC9552 Page 1 of 1

Certificate of Origin

**Product Number:** D8906

Outside USA [eurtechserv@sial.com](mailto:eurtechserv@sial.com)

**Product Name:** Dextran sulfate sodium salt Leuconostoc spp.

#### for molecular biology, average Mw > 500,000 dextran starting material, phosphate buffer 0.5-2 %

**Lot Number:** BCCB4781

**Brand:** Sigma

Country of Manufacture: Denmark

Type of Manufacture

Chemical synthesis / chemical transformation Fermentation / from cell culture Purification/extraction from biological material

Blending of various components (incl. Preparation of solutions)

Purification/extraction from non-biological natural inorganic/organic source

Is the product / material partly or fully of human or animal origin (i.e., tallow derived materials, tissue, tissue extract or fluid such as milk, blood, etc.)?

Does the manufacturing process involve any raw materials, sourcing materials, or reagents that are of human or animal origin (i.e., lubricants, cleaning agents, filtering agents, processing aids, enzymes or cultural media, fermentation)?

Is any of the equipment used for processing and / or storage of the product in contact at any time with other materials of human or animal origin (i.e., cleaning solutions, solutions for equipment, test media used in equipment validation, other human or animal-derived products / materials)?

Is the product / material, for any other reason, at any time during its manufacturing process, in contact with materials of human or animal origin including fermentation?

Does the closure container (e.g. bottles, caps, drum, etc.) involve any raw materials that are of human or animal origin?

Yes Yes No No No

No

No

No

No

No

Is the Product/Material synthetic origin? Yes

Are there procedures in place to avoid cross contamination with other materials or residue of animal, human, GMO origin or Allergen materials?

Is the product/material directly produced through genetic engineering?

**Yes**

No

Genetically modified organisms used No

Have any gluten materials been applied during No manufacturing?

Solvent(s) are used Yes

- Ethanol unknown ppm

During the manufacturing process is a catalyst(s) used? No

Is the product/material directly produced or handled using No ionizing radiation?

During the manufacturing process is a solvent(s) used? Yes

Outside USA [eurtechserv@sial.com](mailto:eurtechserv@sial.com)

Are processes in-place for monitoring the production process, traceability and batch consistency?

Is this product/material collected and/or processed in dedicated equipment?

Was this product/material collected and packaged in new or sterilized containers?

Yes Yes Yes

Soak Yes

Spray Yes

Circulate Yes

Have any allergen materials been applied during No manufacturing?

Biological Origin Fermentation/Cell Culture

Source of fermentation material: microbial

Indicate Species Used Leuconostoc mesenteroides B512F

BSE/TSE statement:

This product-batch was derived from non-animal sources. Therefore this product-batch complied with the requirements stipulated in EMEA/410/01 as **Negligible Risk** for Transmissible Spongiform Ecephalopathy (TSE) /

Bovine Spongiform Ecephalopathy (BSE).

**Document issued by Sigma-Aldrich Corporation "Sigma-Aldrich". This document is valid without signature and has been produced digitally.**

**This information is to be used for the purpose of determining animal or other biological origin only and not to be confused with "country of origin" for import/export purposes. Data provided in this document are property of Sigma-Aldrich.**

**The above information is believed to be correct to the best of our knowledge**

**Sigma-Aldrich shall not be held liable for any damage resulting from the sale, use, handling or from processing the above product(s). This document does not make any warranty, express or implied, of fitness for any particular use of the product(s).**

**Purchaser must determine the suitability of the product(s) for its use under the applicabble laws and regulations.**


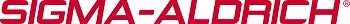

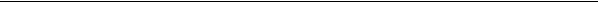


# Certificate of Analysis

3050 Spruce Street, Saint Louis, MO 63103 USA

Email USA: [techserv@sial.com](mailto:techserv@sial.com) Outside USA: [eurtechserv@sial.com](mailto:eurtechserv@sial.com)

#### **Product Name:** Dextran sulfate sodium salt Leuconostoc spp. for molecular biology, average Mw > 500,000

dextran starting material, phosphate buffer 0.5-2 %

**Product Number:** D8906

**Batch Number:** BCCB4781

**Brand:** Sigma

**CAS Number:** 9011-18-1

**Formula:**

**Formula Weight:**

### Storage Temperature: 2-8 C

**Quality Release Date:** 18 JUN 2019

**Recommended Retest Date:** MAR 2023

### TEST SPECIFICATION RESULT

**APPEARANCE (COLOR)** OFF WHITE OFF WHITE

**APPEARANCE (FORM)** POWDER POWDER **SOLUBILITY (COLOR)** COLORLESS TO YELLOW VERY FAINT YELLOW **SOLUBILITY (TURBIDITY)** CLEAR TO SLIGHTLY HAZY CLEAR

**SOLUBILITY (METHOD)** 100 MG/ML IN WATER 100 MG/ML IN WATER

**PH-TEST** 6.0 - 8.0 7.3

**PH (METHOD)** 1 % IN WATER AT 25 C 1 % IN WATER AT 25 C

**LOSS ON DRYING** ≤ 7 % 2 %

**SULFUR CONTENT** 16.0 - 19.0 % (DRY BASIS) 16.9 % (DRY BASIS)

**BIO-TESTS** SUITABLE FOR NUCLEIC ACID HYBRIDIZATION

SUITABLE FOR NUCLEIC ACID HYBRIDIZATION


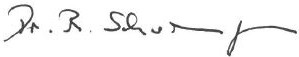


Dr. Reinhold Schwenninger Quality Assurance

Buchs, Switzerland

Sigma-Aldrich warrants that at the time of the quality release or subsequent retest date this product conformed to the information contained in this publication. The current specification sheet may be available at Sigma-Aldrich.com. For further inquiries, please contact Technical Service. Purchaser must determine the suitability of the product

for its particular use. See reverse side of invoice or packing slip for additional terms and conditions of sale.


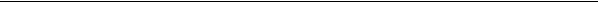


Sigma-Aldrich Certificate of Analysis - Product D8906 Lot BCCB4781 Page 1 of 1


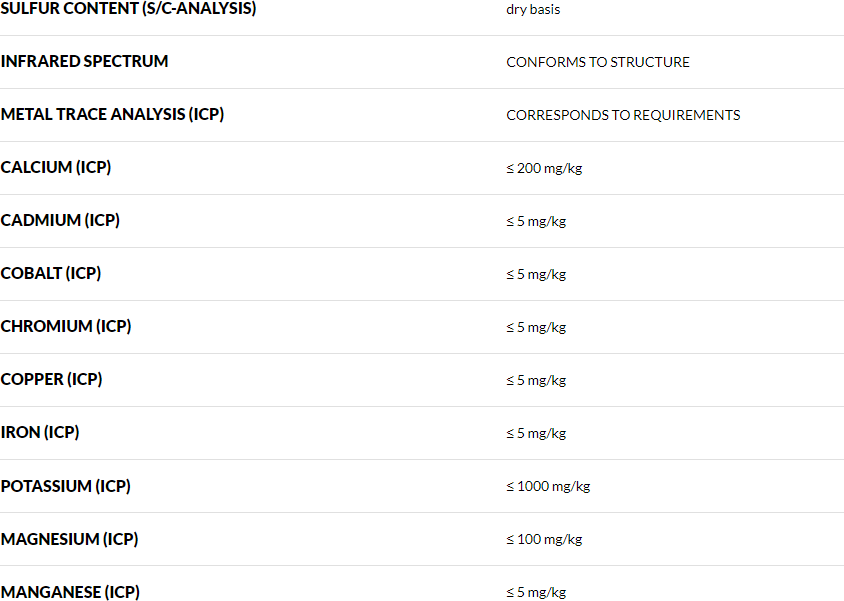

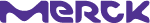


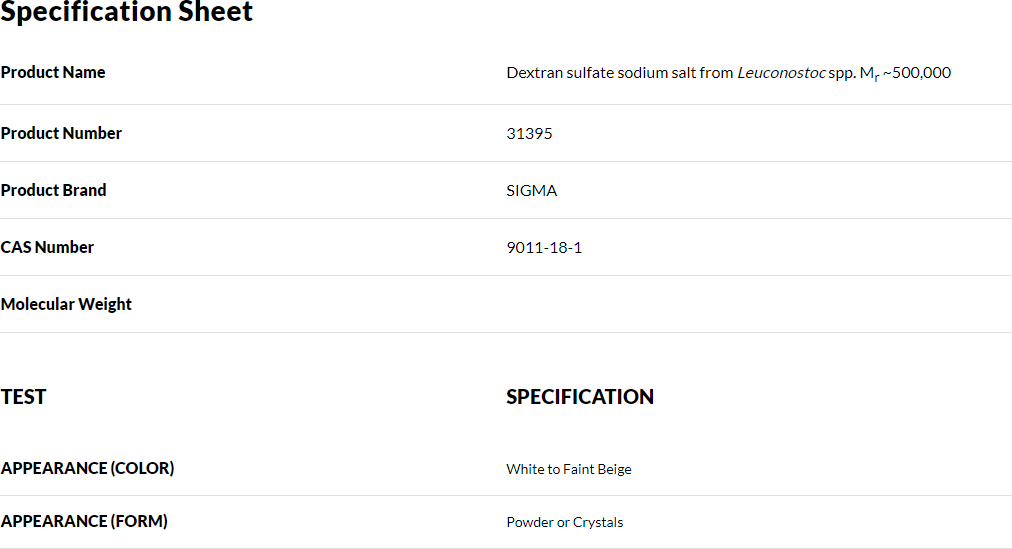


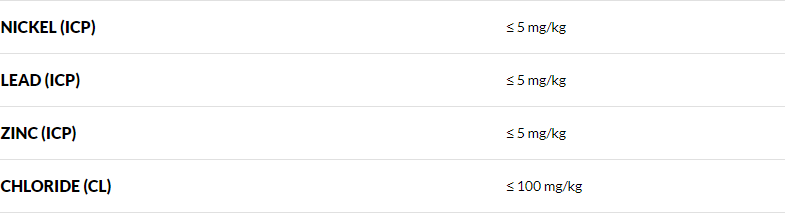


Certificate of Origin

**Product Number:** 51227

**Product Name:** Dextran sulfate sodium salt Mw 7,000-20,000

**Lot Number:** BCCC6032

### Brand:

Country of Manufacture: Japan

Type of Manufacture

Outside USA [eurtechserv@sial.com](mailto:eurtechserv@sial.com)

Chemical synthesis / chemical transformation Fermentation / from cell culture Purification/extraction from biological material

Blending of various components (incl. Preparation of solutions)

Purification/extraction from non-biological natural inorganic/organic source

Is the product / material partly or fully of human or animal origin (i.e., tallow derived materials, tissue, tissue extract or fluid such as milk, blood, etc.)?

Does the manufacturing process involve any raw materials, sourcing materials, or reagents that are of human or animal origin (i.e., lubricants, cleaning agents, filtering agents, processing aids, enzymes or cultural media, fermentation)?

Is any of the equipment used for processing and / or storage of the product in contact at any time with other materials of human or animal origin (i.e., cleaning solutions, solutions for equipment, test media used in equipment validation, other human or animal-derived products / materials)?

Is the product / material, for any other reason, at any time during its manufacturing process, in contact with materials of human or animal origin including fermentation?

Does the closure container (e.g. bottles, caps, drum, etc.) involve any raw materials that are of human or animal origin?

Yes No No No No

No

No

No

No

No

Is the Product/Material synthetic origin? Yes

Are there procedures in place to avoid cross contamination with other materials or residue of animal, human, GMO origin or Allergen materials?

**Yes**

Genetically modified organisms used No

Have any gluten materials been applied during No manufacturing?

Solvent(s) are used Yes

During the manufacturing process is a catalyst(s) used? No

Is the product/material directly produced or handled using No ionizing radiation?

During the manufacturing process is a solvent(s) used? Yes

Are processes in-place for monitoring the production process, traceability and batch consistency?

Is this product/material collected and/or processed in dedicated equipment?

Was this product/material collected and packaged in new or sterilized containers?

Yes Yes Yes

Outside USA [eurtechserv@sial.com](mailto:eurtechserv@sial.com)

Tap Water Yes

Soak Yes

Have any allergen materials been applied during No manufacturing?

BSE/TSE statement:

This product-batch was derived from non-animal sources. Therefore this product-batch is not derived from materials listed in the annex of EMA 410/01/Rev.3 (BSE/TSE guideline).

**Document issued by Sigma-Aldrich Corporation "Sigma-Aldrich". This document is valid without signature and has been produced digitally.**

**This information is to be used for the purpose of determining animal or other biological origin only and not to be confused with "country of origin" for import/export purposes. Data provided in this document are property of Sigma-Aldrich.**

**The above information is believed to be correct to the best of our knowledge**

**Sigma-Aldrich shall not be held liable for any damage resulting from the sale, use, handling or from processing the above product(s). This document does not make any warranty, express or implied, of fitness for any particular use of the product(s).**

**Purchaser must determine the suitability of the product(s) for its use under the applicabble laws and regulations.**


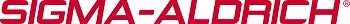

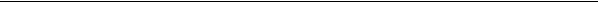


# Certificate of Analysis

**Product Name:** Dextran sulfate sodium salt Mw 7,000-20,000

**Product Number:** 51227

**Batch Number:** BCCC6032

**Brand:** Sigma

### CAS Number:

**Formula:**

### Formula Weight:

**Quality Release Date:** 19 DEC 2019

3050 Spruce Street, Saint Louis, MO 63103 USA

Email USA: [techserv@sial.com](mailto:techserv@sial.com) Outside USA: [eurtechserv@sial.com](mailto:eurtechserv@sial.com)

### TEST SPECIFICATION RESULT

**APPEARANCE (COLOR)** COLORLESS OR WHITE WHITE

**APPEARANCE (FORM)** POWDER POWDER

**GEL-PERMEATION CHROM.** MW: 7000 - 20000 MW: 7243

**SOLUBILITY (COLOR)** COLORLESS TO FAINT GREEN-YELLOW VERY FAINT GREEN-YELLOW

**SOLUBILITY (TURBIDITY)** CLEAR CLEAR

**SOLUBILITY (METHOD)** 500MG IN 5ML WATER 500MG IN 5ML WATER

**LOSS ON DRYING** ≤ 10.0 % 5.8 %

SULFUR CONTENT (S/C-

-ANALYSIS)

17.0 - 19.0 % 18.2 %

**INFRARED SPECTRUM** CONFORMS TO STRUCTURE CONFORMS

**AAS QUALITATIVE** SODIUM CONFIRMED CORRESPONDS


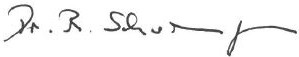


Dr. Reinhold Schwenninger Quality Assurance

Buchs, Switzerland

Sigma-Aldrich warrants that at the time of the quality release or subsequent retest date this product conformed to the information contained in this publication. The current specification sheet may be available at Sigma-Aldrich.com. For further inquiries, please contact Technical Service. Purchaser must determine the suitability of the product

for its particular use. See reverse side of invoice or packing slip for additional terms and conditions of sale.


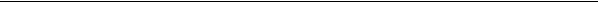


Sigma-Aldrich Certificate of Analysis - Product 51227 Lot BCCC6032 Page 1 of 1

Certificate of Origin

**Product Number:** 42867

**Product Name:** Dextran sulfate sodium salt Mr ~ 40,000

**Lot Number:** BCCB5021

**Brand:** Sigma

Country of Manufacture: Sweden

Type of Manufacture

Outside USA [eurtechserv@sial.com](mailto:eurtechserv@sial.com)

Chemical synthesis / chemical transformation Fermentation / from cell culture Purification/extraction from biological material

Blending of various components (incl. Preparation of solutions)

Purification/extraction from non-biological natural inorganic/organic source

Is the product / material partly or fully of human or animal origin (i.e., tallow derived materials, tissue, tissue extract or fluid such as milk, blood, etc.)?

Does the manufacturing process involve any raw materials, sourcing materials, or reagents that are of human or animal origin (i.e., lubricants, cleaning agents, filtering agents, processing aids, enzymes or cultural media, fermentation)?

Is any of the equipment used for processing and / or storage of the product in contact at any time with other materials of human or animal origin (i.e., cleaning solutions, solutions for equipment, test media used in equipment validation, other human or animal-derived products / materials)?

Is the product / material, for any other reason, at any time during its manufacturing process, in contact with materials of human or animal origin including fermentation?

Does the closure container (e.g. bottles, caps, drum, etc.) involve any raw materials that are of human or animal origin?

Yes No No No No

No

No

No

No

No

Is the Product/Material synthetic origin? Yes

Are there procedures in place to avoid cross contamination with other materials or residue of animal, human, GMO origin or Allergen materials?

Is the product/material directly produced through genetic engineering?

**Yes**

No

Genetically modified organisms used No

Have any gluten materials been applied during No manufacturing?

Solvent(s) are used Yes

During the manufacturing process is a catalyst(s) used? No

Is the product/material directly produced or handled using No ionizing radiation?

During the manufacturing process is a solvent(s) used? Yes

Are processes in-place for monitoring the production process, traceability and batch consistency?

Is this product/material collected and/or processed in dedicated equipment?

Yes No

Outside USA [eurtechserv@sial.com](mailto:eurtechserv@sial.com)

Was this product/material collected and packaged in new or sterilized containers?

**Yes**

Tap Water Yes

Distilled Water Yes

Detergent Yes

Caustic Yes

Soak Yes

Spray Yes

Have any allergen materials been applied during No manufacturing?

BSE/TSE statement:

This product-batch was derived from non-animal sources. Therefore this product-batch complied with the requirements stipulated in EMEA/410/01 as **Negligible Risk** for Transmissible Spongiform Encephalopathy (TSE) /

Bovine Spongiform Encephalopathy (BSE).

**Document issued by Sigma-Aldrich Corporation "Sigma-Aldrich". This document is valid without signature and has been produced digitally.**

**This information is to be used for the purpose of determining animal or other biological origin only and not to be confused with "country of origin" for import/export purposes. Data provided in this document are property of Sigma-Aldrich.**

**The above information is believed to be correct to the best of our knowledge**

**Sigma-Aldrich shall not be held liable for any damage resulting from the sale, use, handling or from processing the above product(s). This document does not make any warranty, express or implied, of fitness for any particular use of the product(s).**

**Purchaser must determine the suitability of the product(s) for its use under the applicabble laws and regulations.**


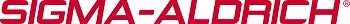

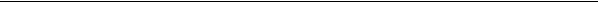


# Certificate of Analysis

3050 Spruce Street, Saint Louis, MO 63103 USA

Email USA: [techserv@sial.com](mailto:techserv@sial.com) Outside USA: [eurtechserv@sial.com](mailto:eurtechserv@sial.com)

**Product Name:** DEXTRAN SULFATE SODIUM SALT Mr ~ 40,000

**Product Number:** 42867

**Batch Number:** BCCB5021

**Brand:** Sigma

### CAS Number:

**Formula:**

### Formula Weight:

**Quality Release Date:** 24 APR 2019

### TEST SPECIFICATION RESULT

**APPEARANCE (COLOR)** WHITE TO OFF WHITE WHITE

**APPEARANCE (FORM)** POWDER POWDER

MOLECULAR WEIGHT DETERMINATION

WEIGHT AVERAGE MOLECULAR WEIGHT (MW) ~ 40000

MW: 47739

**LOSS ON DRYING** ≤ 10 % 4 %

SULFUR CONTENT (S/C-

-ANALYSIS)

15.0 - 19.0 18 %


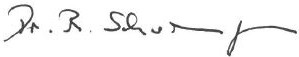


Dr. Reinhold Schwenninger Quality Assurance

Buchs, Switzerland

Sigma-Aldrich warrants that at the time of the quality release or subsequent retest date this product conformed to the information contained in this publication. The current specification sheet may be available at Sigma-Aldrich.com. For further inquiries, please contact Technical Service. Purchaser must determine the suitability of the product

for its particular use. See reverse side of invoice or packing slip for additional terms and conditions of sale.


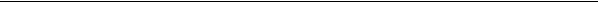


Sigma-Aldrich Certificate of Analysis - Product 42867 Lot BCCB5021 Page 1 of 1

**B**


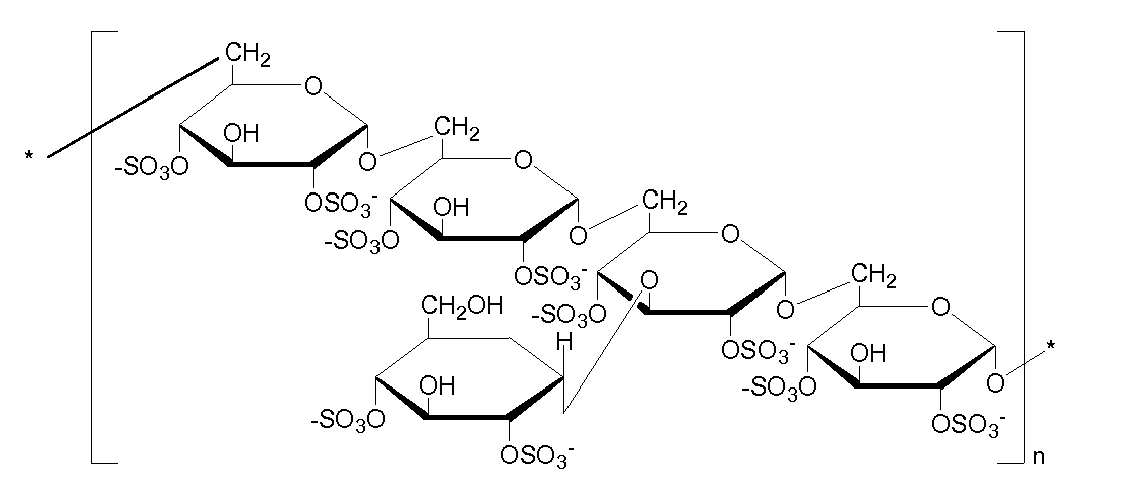


**Figure S1**. **A)** Certificate of analysis and origin of the compounds chondroitin sulfate sodium salt from shark cartilage (P1), dextran sulfate sodium salt from *Leuconostoc mesenteroides* B512F M_w_ >500,000 Da (P2), dextran sulfate sodium salt M_w_ 7,000-20,000 Da (P3), and dextran sulfate sodium salt M_r_ ~40,000 Da (P4) provided by SIGMA-ALDRICH. **B)** Molecular structure of dextran sulfate sodium salt.

**S2.**


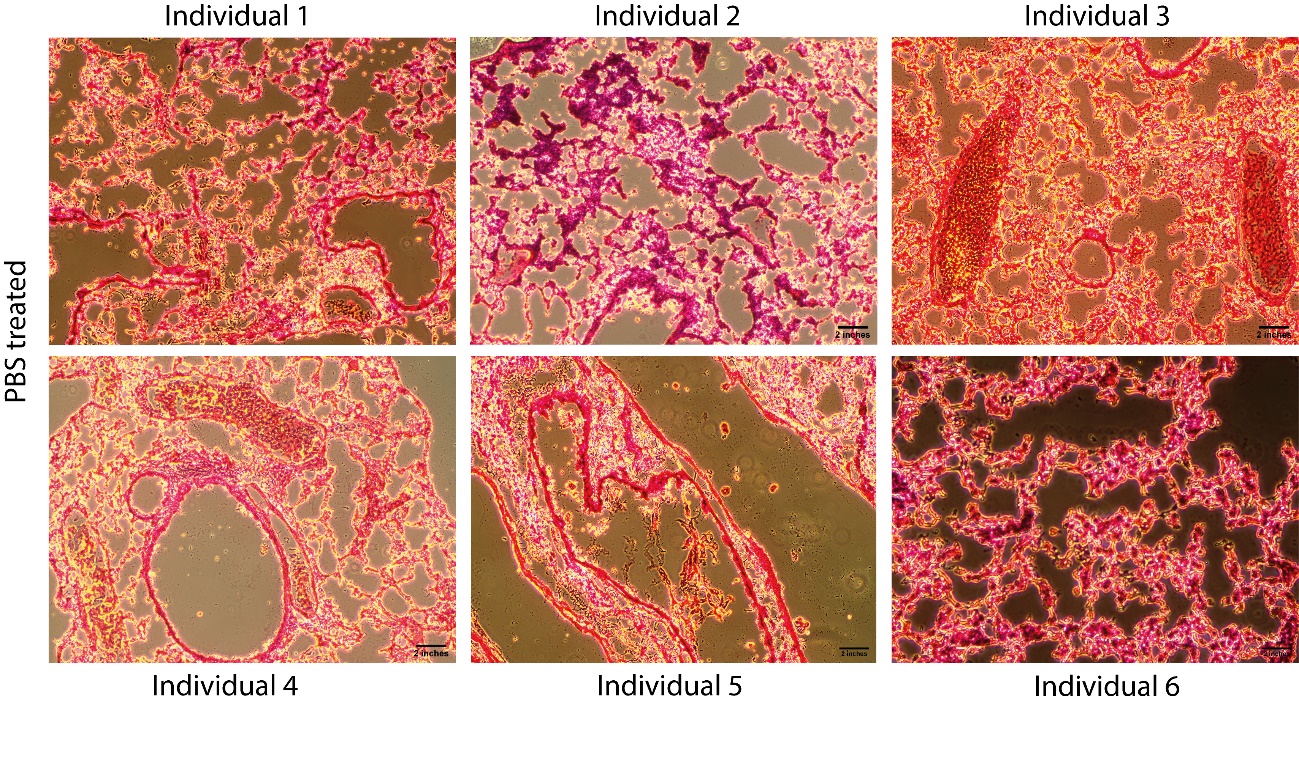


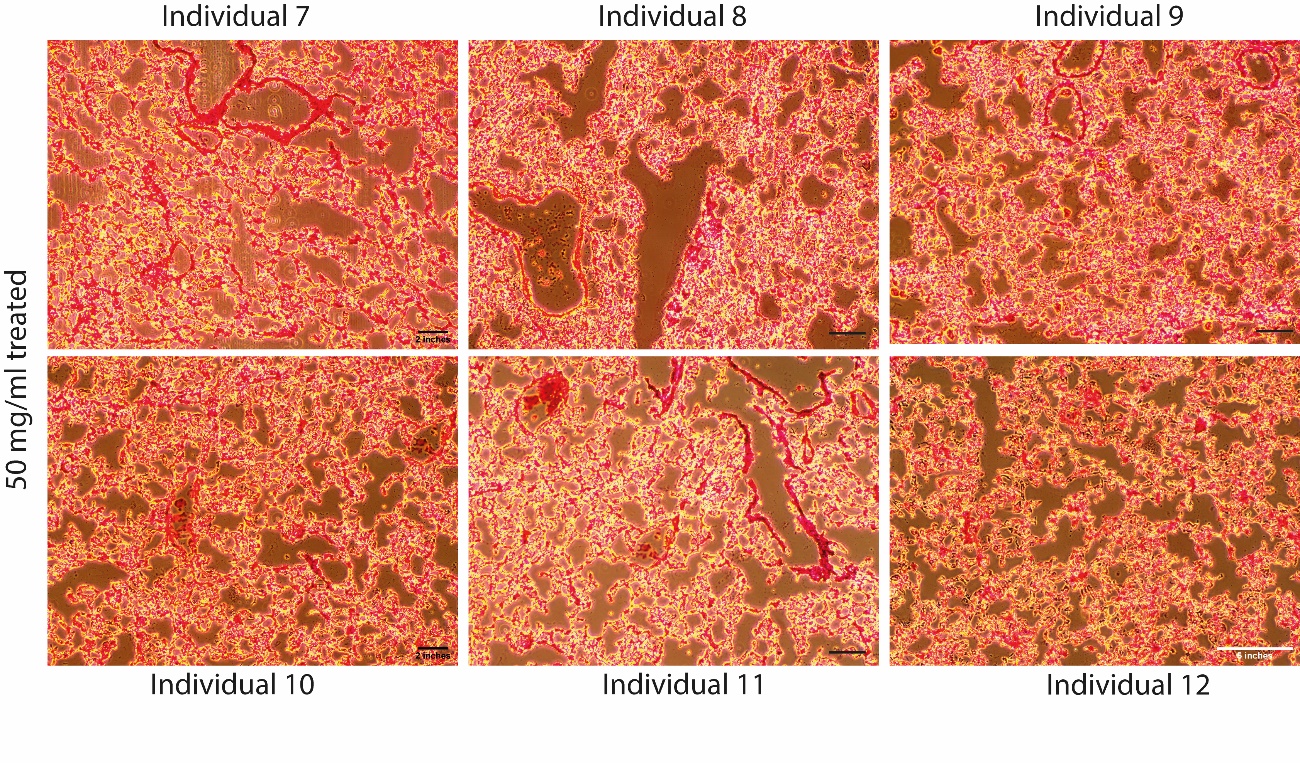


**Figure S2.** Hematoxylin and eosin (H&E) staining of a SARS-CoV-2 infected mice lung tissue 8 days p.i (scale bar, 100 μm, *n*=6), treated with either PBS or 50 mg/ml P2. Representative cryosections from each individual are shown.
